# Repurposing HFC-125 to tetrafluoroethylene: A step toward a more sustainable fluoropolymer feedstock strategy

**DOI:** 10.1016/j.isci.2025.112580

**Published:** 2025-05-03

**Authors:** Hiroto Iwasaki, Naoyuki Hoshiya, Yosuke Kishikawa, Jorge Escorihuela, Norio Shibata

**Affiliations:** 1 Department of Nanopharmaceutical Sciences, Nagoya Institute of Technology, Gokiso, Showa-ku, Nagoya 466-8555, Japan; 2 Department of Life Science and Applied Chemistry, Nagoya Institute of Technology, Gokiso, Showa-ku, Nagoya 466-8555, Japan; 3 Technology and Innovation Center, DAIKIN Industries, Ltd, 1-1 Nishi-Hitotsuya, Settsu, Osaka 566-8585, Japan; 4 Departamento de Química Orgánica, Universitat de València, Avda. Vicente Andrés Estellés s/n, Burjassot 46100 Valencia, Spain; 5 Instituto de Ciencia Molecular (ICMol), Universitat de València, Calle Catedrático José Beltrán 2, Paterna, Valencia, Spain

**Keywords:** Chemistry, Green chemistry

## Abstract

The urgency to reduce hydrofluorocarbon (HFC) emissions, particularly HFC-125 (pentafluoroethane, CF_3_CF_2_H), has driven efforts to develop sustainable alternatives. Herein, we present a method for converting HFC-125 into tetrafluoroethylene (TFE), an industrially valuable monomer for fluoropolymer production. Our approach uses potassium hexamethyldisilazide (KHMDS), optimizing reaction conditions at −50°C to achieve high TFE yields without any observable byproducts. This low-temperature method offers a safer and more sustainable alternative to traditional high-temperature processes for TFE production, which involve hazardous byproducts and complex handling. We also demonstrated that various chemical reactions using freshly generated TFE, including thiol addition, trifluorovinylation, radical addition, amination, alcohol addition, and polymerization, can be performed, extending the utility of this approach. Moreover, a continuous flow process for the conversion of HFC-125 to TFE at room temperature was achieved without cryogenic equipment. This dual-purpose solution addresses both environmental sustainability of fluorochemicals and the growing demand for fluoropolymers in various industries.

## Highlights

• Selective dehydrofluorination based on negative hyperconjugation
• Synthesis of tetrafluoroethylene from the pentafluoroethane
• Continuous flow tetrafluoroethylene synthesis at room temperature
• Potassium cation promotes β-fluorine elimination

## Graphical abstract

**Figure:**
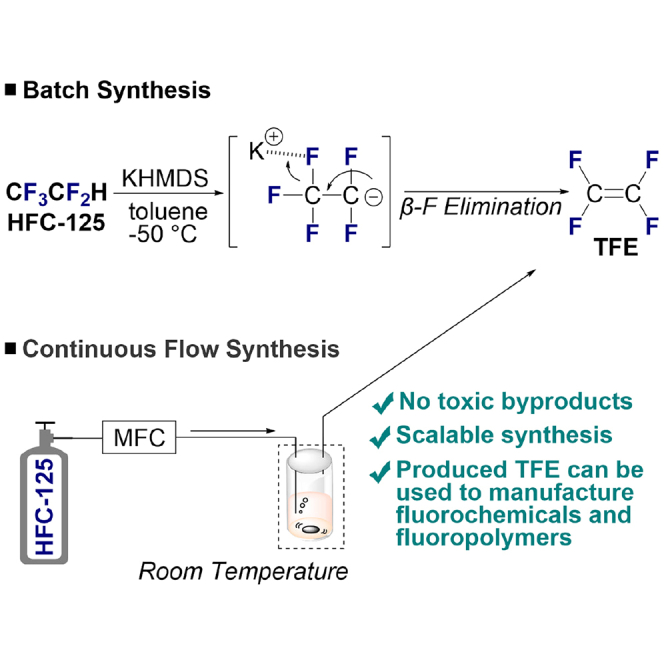

## Introduction

The quest to replace chlorofluorocarbons (CFCs) and hydrochlorofluorocarbons (HCFCs) has led to the development and widespread use of hydrofluorocarbons (HFCs), which were initially hailed as safer alternatives because of their negligible ozone depletion potential (ODP). Among these, HFC-125 (pentafluoroethane, CF_3_CF_2_H) has become prominent in applications such as fire extinguishing agents and refrigerant blends like HFC-410A.^1^^,^^2^^,^^3^^,^^4^ However, the early optimism surrounding HFCs has been tempered by their high global warming potential (GWP), with HFC-125 having a GWP of 3,450.^5^ The 2016 Kigali Amendment to the Montreal Protocol was pivotal, marking the recognition of the climate impact posed by HFCs and mandating their phase-down.^6^^,^^7^^,^^8^^,^^9^^,^^10^ In response to these regulatory strides, the urgency to develop more effective and scalable methods for the decomposition of HFC has never been greater, not only just as a matter of environmental stewardship but also as a necessity for mitigating future climate impacts.

**Figure 1:**
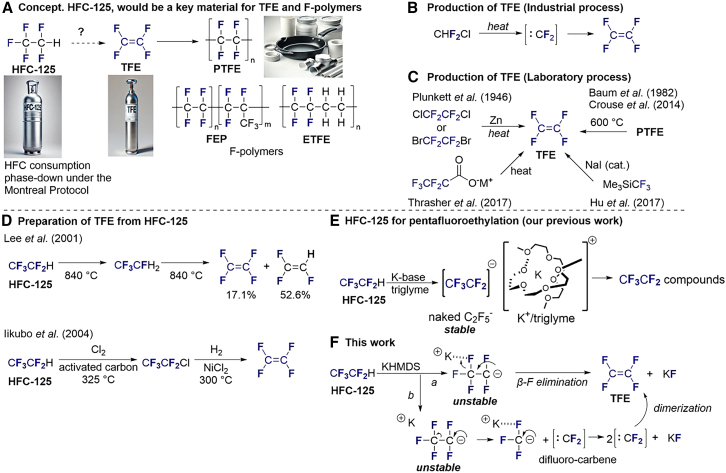
Background and challenges (A) Concept, HFC-125 to F-polymers via TFE. (B and C) Synthesis of TFE (industrial method B and laboratory methods C). (D) Previous synthetic attempts to convert HFC-125 to TFE. (E) Our previous work. (F) A proof of concept. Transformation of HFC-125 to TFE (This work).

An ideal approach to address this challenge involves the transformation of HFC-125 into tetrafluoroethylene (TFE, CF_2_ = CF_2_), a key monomer in the production of fluoropolymers, such as polytetrafluoroethylene (PTFE), ethylene tetrafluoroethylene (ETFE), and fluorinated ethylene propylene (FEP) (Figure 1A). TFE is particularly valued for its negligible GWP and ODP,^11^ making it an environmentally favorable compound in the chemical industry. Traditionally, TFE has been produced by the dimerization of difluorocarbenes (CF_2_) generated by the pyrolysis of chlorodifluoromethane (R-22) (Figure 1B).^12^^,^^13^^,^^14^^,^^15^^,^^16^^,^^17^^,^^18^^,^^19^ This method, while industrially effective, generates hazardous byproducts, such as hydrogen chloride (HCl) and perfluoroisobutene,^20^^,^^21^^,^^22^^,^^23^^,^^24^ necessitating complex purification and making it less viable for smaller-scale applications. Other industrial methods, such as plasma,^25^^,^^26^ microwave,^25^ and pyrolysis (at 600–1,000 K)^27^ degradation of perfluoroalkenes to TFE, have been found to be inefficient. Several laboratory methods have been used for the synthesis of TFE (Figure 1C), such as the dechlorination of 1,2-dichloro-1,1,2,2-tetrafluoroethylene using zinc,^28^^,^^29^^,^^30^ pyrolysis of pentafluoropropionic acid salts,^31^^,^^32^ decomposition of PTFE,^33^^,^^34^^,^^35^^,^^36^^,^^37^^,^^38^^,^^39^^,^^40^ or others.^41^^,^^42^^,^^43^^,^^44^^,^^45^^,^^46^^,^^47^ Recently, Hu et al. reported the synthesis of TFE from the Ruppert-Prakash reagent (Me_3_SiCF_3_) in the presence of sodium iodide through the generation of CF_2_.^48^ However, these synthetic approaches are not viable on an industrial scale. As the global fluoropolymer market is projected to grow at a compound annual growth rate (CAGR) of approximately 6% between 2023 and 2033,^49^ driven by demand from the automotive, electronics, and renewable energy sectors, the need for more sustainable and scalable production of TFE is evident.^50^^,^^51^^,^^52^^,^^53^^,^^54^ This demand, coupled with the environmental imperative for managing HFC-125, presents a compelling case for exploring the transformation of HFC-125 into TFE as a dual-purpose solution that addresses both environmental and industrial needs (Figure 1A).

Historically, the conversion of HFC-125 to TFE has been explored but with limited success (Figure 1D). In 2001, Lee et al. demonstrated a method for producing aliphatic fluorocarbons through the pyrolysis of fluorocarbons,^55^ in which HFC-125 was converted to R-134a and subsequently to trifluoroethylene and TFE, albeit in minimal yield. In 2004, Iikubo et al. developed a complex pathway involving the conversion of HFC-125 to chloropentafluoroethane (CFC-115) before generating TFE and hexafluoropropylene (HFP).^56^ Although these methods provide proof of concept as shown in Figure 1A, they are hindered by the need for high temperatures and multiple steps, limiting their practicality for industrial scaling. Recognizing these limitations, our study seeks to pioneer a more efficient one-step process for converting HFC-125 to TFE. Previously, our group reported the successful pentafluoroethylation of carbonyl compounds^57^^,^^58^^,^^59^ and imines^60^ using HFC-125 in conjunction with potassium bases (Figure 1E). The core of this success stems from the strategic use of potassium cations (K^+^) in a triglyme medium, which effectively stabilizes the highly reactive potassium pentafluoroethyl anion (K^+^/CF_3_CF_2_^−^) by encapsulating K^+^, thereby preventing its premature decomposition. Building upon this insight, we have now employed an inverse strategy: intentionally promoting the decomposition of the pentafluoroethyl anion (CF_3_CF_2_^−^) through selective β-fluorine elimination driven by negative hyperconjugation.^61^ This process efficiently generates the target compound, TFE, along with potassium fluoride (KF) (Figure 1F, *route a*). Alternatively, a stepwise mechanism (*route b*) may also occur, involving the decomposition of CF_3_CF_2_^−^ into an unstable trifluoromethyl anion (CF_3_^−^) and CF_2_. The trifluoromethyl anion then further decomposes into CF_2_ and KF, followed by the dimerization of CF_2_ to yield TFE. This innovative approach not only provides an environmentally responsible solution for converting HFC-125 but also supports the growing demand for sustainable fluoropolymer production^50^^,^^62^^,^^63^^,^^64^^,^^65^^,^^66^^,^^67^^,^^68^^,^^69^^,^^70^^,^^71^^,^^72^^,^^73^^,^^74^^,^^75^^,^^76^ (Figure 1A).

## Results and discussion

### Optimization of decomposition conditions

**Figure 2:**
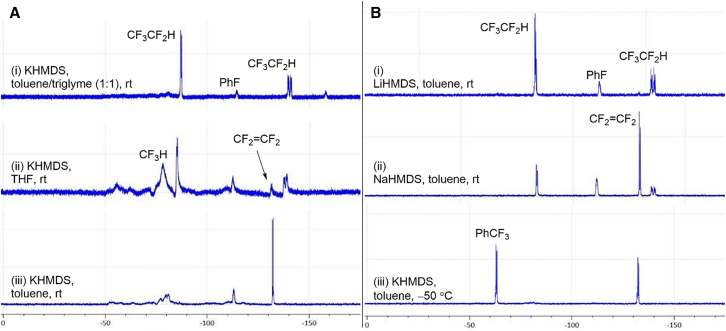
^19^F NMR chart for each decomposition condition of HFC-125 (A) Optimization of the solvent using KHMDS. (B) Optimization of different metal cations and reaction temperatures in toluene.

To investigate the transformation process, we began our investigations by conducting a series of ^19^F NMR experiments at room temperature using HFC-125 (0.6 mmol) and KHMDS (1.0 equiv, 0.6 mmol) in a toluene/triglyme (1:1) solvent mixture for 1 h (Figure 2A [i]). Consistent with our previous studies on pentafluoroethylation,^57^^,^^59^ by the formation of stable CF_3_CF_2_^−^ in triglyme, only intact HFC-125 peaks were detected, with no signs of decomposition. When THF was used as the solvent, we detected a minor signal for TFE, accompanied by the formation of fluoroform (CF_3_H) (Figure 2A [ii]), which might suggest the potential “*route a*” for formation of TFE (Figure 1F, *route b*). On the contrary, when toluene was used, the desired TFE was clearly observed, with the HFC-125 signals disappearing almost completely (Figure S1), although traces of CF_3_H were present (Figure 2A [iii]). Encouraged by these results, we examined the influence of different metal bases. It was found that NaHMDS also facilitated TFE formation, but the residual HFC-125 was still detected (Figure 2B [i]). In contrast, no TFE formation was observed when LiHMDS was used as a base (Figure 2B [ii]). After a 12 h reaction, TFE was detected, though a substantial 70% of HFC-125 remained unreacted (Figure S2). Thus, KHMDS in toluene is more suitable for this synthetic transformation (Figure 2A [iii]). In particular, performing the reaction at −50°C for 3 h (Figure S3) significantly improved the TFE production, eliminating CF_3_H formation and resulting in the visible precipitation of KF (Figure 2B [iii]).

With these optimized conditions, we further refined the reaction using *d8*-toluene at −50°C and varying equivalents of KHMDS. Calibration curves generated with pure gaseous TFE (see Figure S4) allowed accurate quantification of the TFE yield (Table 1). Under the initial conditions (0.025 mmol HFC-125 and 1.0 equiv KHMDS), the TFE yield was 27%, with 30% of HFC-125 remaining unreacted (entry 1). Increasing KHMDS to 1.5 equivalents improved the yield to 64%, with no detectable HFC-125 in the nuclear magnetic resonance (NMR) spectra. This result was consistently reproducible in three additional trials, with yields averaging approximately 70%.

**Table 1:** Determination of the TFE yield based on the calibration curve


Entry^a^	KHMDS (X equiv)	Integral value		Yield (%)^b^	
		HFC-125	TFE	HFC-125	TFE
1	1.0	37.31	30.20	30	27
2	1.5	0	71.30	0	64
3	1.5	0	83.68	0	75
4	1.5	0	69.99	0	63
5	1.5	0	79.90	0	71
6	2.0	0	64.44	0	58
7	2.0	0	88.48	0	79
8	2.0	0	85.89	0	77
9	2.0	0	72.65	0	65
10	3.0	0	58.25	0	52
11^c^	1.5	128.78	0	>99	0
12^d^	1.5	73.04	48.17	57	43

a Reaction was performed using 0.025 mmol of HFC-125.

b Yield was determined by^19^F NMR using C_6_F_6_ as the internal standard.

c Reaction was performed using LiHMDS.

d Reaction was performed using NaHMDS.

Further increasing KHMDS to 2.0 equiv gave a similar yield range (58–79%, four trials), while using 3.0 equiv resulted in a decrease in yield of 52% (entry 10). In all cases, HFC-125 was fully consumed, with the maximum TFE yield reaching 80%. We hypothesized that the lower boiling point of TFE compared to HFC-125 may lead to some loss during NMR analysis, suggesting that the transformation of HFC-125 to TFE likely proceeds near quantitatively. Finally, we reassessed the effect of different metal cations (entries 11 and 12), confirming that potassium cation is the most effective in promoting the transformation of HFC-125 to TFE, in agreement with our initial NMR observations (Figure 2B).

The mechanism for the conversion of HFC-125 to TFE can be explained by two possible pathways (Figure 1F). The first involves direct β-fluorine elimination from CF_3_CF_2_^−^, driven by negative hyperconjugation (*route a*). The second is a stepwise process (*route b*), where CF_2_ is formed, followed by its dimerization into TFE. Our ^19^F NMR experiments detected the formation of CF_3_H alongside TFE, indicating that CF_2_ generation is likely involved. However, dimerization of CF_2_ to form TFE typically requires elevated temperatures, whereas our optimized protocol operates at much lower temperatures, down to −50°C. This suggests that the transformation of HFC-125 to TFE at low temperatures is unlikely to proceed through the CF_2_ dimerization pathway (*route b*). Instead, the generated CF_2_ may be contributing to the formation of unidentified by-products observed in ^19^F NMR experiments at room temperature (Figure 2A). Importantly, the ^19^F NMR spectra recorded at −50°C show only the formation of TFE, with no signals corresponding to by-products (Figure 2B [iii]). Therefore, we conclude that the conversion of HFC-125 to TFE occurs predominantly via direct β-fluorine elimination (*route a*).

### Mechanism study of TFE formation by DFT calculation

**Figure 3:**
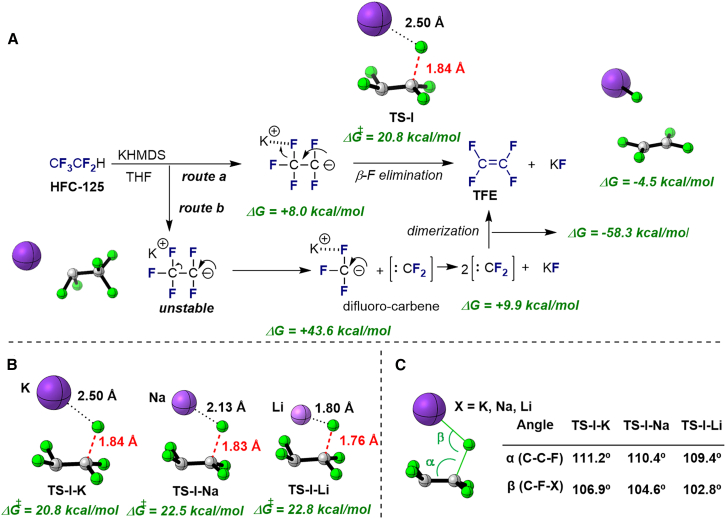
DFT studies (A) Proposed mechanistic pathway based on DFT calculations (Gibbs energy values given at 298.15 K). (B) Optimized structures for TS-I based on different metal bases: KHMDS, NaHMDS, LiHMDS. (C) Structural parameters of the different transition states.

To further elucidate the mechanism of TFE formation (Figure 3A), density functional theory (DFT) calculations were performed using the Gaussian 16 package at the wB97XD/def2TZVP level of theory with the conductor-like polarizable continuum model (CPCM) to mimic the effect of the solvent (toluene). The first step involves the deprotonation of HFC-125 to form the unstable C_2_F_5_^−^ anion. While this process is slightly endergonic (+8.0 kcal/mol), the energy values are reasonable for the reaction to proceed under the strongly basic conditions provided by KHMDS. In the β-fluorine elimination pathway from the C_2_F_5_^−^ anion to TFE, facilitated by KF (*route a*), the reaction is slightly exergonic (−4.5 kcal/mol) and proceeds through a transition state (TS-I) an overall activation barrier of 20.8 kcal/mol. The computed activation barrier in THF was 22.4 kcal/mol, in agreement with the lower conversion in that solvent. The computed barrier in the absence of K^+^ cation, i.e., when using triglyme was increased up to 25.4 kcal/mol, being consistent with the experimental observation of no decomposition HFC-125. These results suggest that *route a* is plausible based on DFT calculations. On the other hand, *route b*, which involves the generation of a CF_3_^−^ anion and CF_2_, followed by dimerization of CF_2_ to form TFE, is less favorable. According to the DFT calculations, the transition state of the decomposition of C_2_F_5_^−^ anion into CF_3_^−^ and CF_2_ could not be identified and thus *route b* was not favorable. Although we did not anticipate that the dimerization of CF_2_ to TFE would be highly exergonic (−58.3 kcal/mol) and feasible, the initial step of generating CF_3_^−^ and CF_2_ is highly endergonic (+43.6 kcal/mol), making this pathway less likely. The subsequent step of generating CF_2_ from CF_3_^−^ is also endergonic (+9.8 kcal/mol), making this pathway less likely Nevertheless, the detection of CF_3_H in the NMR studies suggests that *route b* may occur as a minor process. DFT calculations also support the formation of CF_3_H from CF_3_^−^ anion with an exergonicity of 10.6 kcal/mol.

As mentioned previously, NaHMDS also facilitated TFE formation, but no TFE formation occurred when LiHMDS was used as a base. Similar Gibbs energies were computed for the first step involving the deprotonation of HFC-125 to form the unstable C_2_F_5_^−^ anion. This process was found to be also endergonic (+8.6 kcal/mol) under NaHMDS, while 11.3 kcal/mol were computed for LiHMDS. Higher activation barriers for the β-fluorine elimination yielding TFE compared to that of KHMDS were computed when using NaHMDS and LiHMDS, with values of 22.5 and 22.8 kcal/mol, respectively (Figure 3B). A closer look at the transition state structures reveals structural differences on the optimized structures. As the cation size increases from Li to K, the C-C-F (α) and C-F-X (β) angles also increase, which may facilitate the fluorine elimination (Figure 3C). These results show that while all reactions are thermodynamically feasible, the activation barriers differ, with KHMDS facilitating the fastest reaction.

### Application of generated TFE

**Figure 4:**
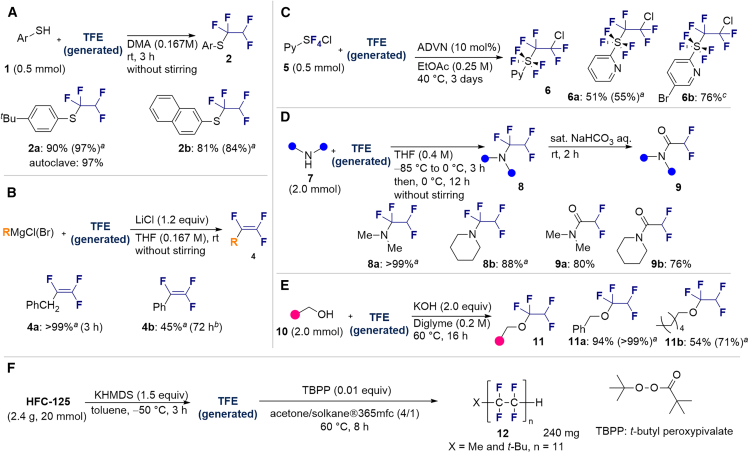
Chemical reaction using generated TFE (A) Hydrothiolation. (B) Trifluoro-vinylation with RMgX. (C) Addition of PySF_4_Cl. (D) Amination. (E) Alkoxylation. (F) Polymerization. ^a^Yield is in parentheses, determined by ^19^F-NMR using PhCF_3_ as an internal standard. ^*b*^Reaction was performed using PhMgBr (0.6 mmol), HFC-125 (8.3 equiv), KHMDS (12.5 equiv). ^*c*^Reaction was performed using **5b** (0.6 mmol), HFC-125 (5.0 equiv), KHMDS (7.5 equiv).

With the successful transformation of HFC-125 into TFE, we explored various chemical reactions using freshly generated TFE within a Schlenk tube. Our first investigation focused on the addition of arylthiols **1** to TFE following the Ogoshi protocol^77^ (Figure 4A). TFE (6.0 equivalents, generated from 3.0 mmol of HFC-125) was transferred to a separate Schlenk tube containing a DMA solution of **1** under a liquid-nitrogen-cooled vessel and allowed to react at room temperature for 3 h. This procedure provided the corresponding thiol adducts **2** in excellent yields (**2a**: 90%, **2b**: 81%). To monitor the pressure changes during the reaction accurately, we repeated the synthesis of **2a** in an autoclave with arylthiol **1a**. The pressure gauge showed a maximum increase of 0.1 MPa, which gradually returned to atmospheric pressure after 3 h, resulting in a 97% yield of **2a**.

Next, we turned our attention to the selective trifluorovinylation^78^ of alkyl and aryl Grignard reagents **3** (RMgCl, Figure 4B). Benzyl-MgCl (**3a**) was treated with TFE (generated from HFC-125) at room temperature for 3 h without stirring, producing (2,3,3-trifluoroallyl) benzene (**4a**) quantitatively, as confirmed by ^19^F NMR analysis. When PhMgBr (**3b**) was employed, α, β, β-trifluorostyrene (**4b**) was obtained in 45% yield after allowing the reaction to proceed at room temperature for 72 h. Our observed yields are consistent with previous reports.^78^ The higher reactivity of **3a** compared to **3b** can be attributed to the partial resonance stabilization of the transition state, which facilitates the trifluorovinylation reaction.

We also explored the radical addition reaction between TFE and *o*-pyridinyl-tetra-fluorosulfanyl chlorides (PySF_4_Cl, **5**)^79^^,^^80^^,^^81^ (Figure 4C), using the method reported by Nozaki et al. in 2023.^82^ TFE, generated from 2 mmol HFC-125, was transferred to a solution of PySF_4_Cl **5a** and 10 mol % ADVN, and the mixture was stirred at 40°C in ethyl acetate for 3 days. This reaction yielded the adduct **6a** in 51%. The bromide moiety in Br-PySF_4_Cl (**5b**) was well tolerated under the conditions, providing tetrafluoroethyl addition product **6b** in 76% yield.

To further extend the utility of TFE, we scaled up its generation and explored amination reactions^83^^,^^84^ in an autoclave. TFE, produced from 10 mmol of HFC-125, was transferred to a THF solution containing 2.0 mmol of amine **7** at −85°C. The reaction mixture was gradually warmed to 0°C and left to stand for 12 h without stirring. When dimethylamine (**7a**) was used, the reaction proceeded quantitatively, yielding 1,1,2,2-tetrafluoro-N,N-dimethylethylamine (TFEDMA, **8a**). The treatment of **8a** with sat. NaHCO_3_ aq. afforded the corresponding amide derivative **9a** in 80% yield. A similar reaction with piperidine (**7b**) produced piperidine-tetrafluoroethyl adduct **8b** in 88% yield, which upon hydrolysis gave the amide **9b** in 76% yield (Figure 4D).

Moreover, we examined the addition of alcohols^85^^,^^86^ to TFE (Figure 4E). Using a scale similar to that used for the amination reaction (Figure 4D), TFE was transferred to a diglyme solution containing 2.0 equiv of KOH and alcohols **10** (2.0 mmol) and stirred at 60°C for 16 h. The reaction with benzyl alcohol (**10a**) proceeded efficiently, delivering desired benzyl tetrafluoroethyl ether (**11a**) in 94% isolated yield. In the case of 1-hexanol (**10b**), the corresponding tetrafluoroethyl ether **11b** was obtained in a moderate yield of 54% (isolated). The difference in yields between **10a** and **10b** can be explained by the higher nucleophilicity of **10a**. The electron-donating nature of the benzyl group enhances nucleophilicity, leading to a more efficient alkoxylation process. These observations align well with previously reported findings.^85^^,^^86^

The radical polymerization^87^ of the generated TFE was initiated by *tert-*butyl peroxypivalate (TBPP) and conducted at 60°C for 8 h in acetone/solkane 365mfc within an autoclave. This process yielded 240 mg of the desired TFE polymer, X-CF_2_-(CF_2_-CF_2_)_n_-CF_2_-H (**12**, *n* = 11, X = Me and *t*-Bu), from 2.4 g of HFC-125 (Figure 4F). The ^19^F NMR spectrum closely matches that of an authentic PTFE sample, displaying a characteristic signal at −122 ppm along with three additional spinning side bands (Figure S5). Two additional terminal peaks were observed at −113 ppm (assigned to X-CF_2_-, where X = Me and *t*-Bu) and −138 ppm (assigned to –CF_2_H). The degree of polymerization (*n* = 11) was determined by comparing the integration of the main CF_2_ peak (−122 ppm) with the terminal CF_2_H peak (Figure S6).

### Continuous flow synthesis of TFE

Finally, we sought to extend our bath transformation method for HFC-125 to TFE in a continuous flow process. The continuous flow approach offers significant advantages for large-scale chemical transformations, particularly when working with gaseous substrates,^88^^,^^89^^,^^90^ without requiring substantial modifications to reactor size. This method enables improvements in reaction conditions, such as drastically reduced reaction times and the elimination of cryogenic temperatures. Furthermore, it facilitates seamless scalability through continuous operation or parallelization, making it an ideal strategy for large-scale industrial applications.

We initially attempted the transformation of HFC-125 into TFE using a conventional micromixer, as employed in our previous work.^58^^,^^59^^,^^60^^,^^91^ However, this approach led to clogging of the flow path due to the precipitation of KF. To address this issue, we designed a new device in which HFC-125 is continuously bubbled into a KHMDS solution in toluene at room temperature while being vigorously stirred. The generated TFE is then continuously transferred into a nearby collector maintained at −85°C (Table 2). The flow rate of HFC-125 was controlled at 3.35 mL/min using a mass flow controller, while KHMDS, corresponding to 4.0 equivalents of HFC-125, was bubbled into a 1.0 M toluene solution. After bubbling for 5 min, resulting in an outflow of 0.75 mmol of HFC-125, the ^19^F NMR spectrum of the collected gas revealed no trace of HFC-125, with TFE obtained in 77% yield (entry 1). When the bubbling time was extended to 10 min (HFC-125: 1.50 mmol) with the same amount of KHMDS as in entry 1, 40% of TFE was produced, and 35% of HFC-125 remained (entry 2). Subsequently, increasing the amount of KHMDS to 3.0 equivalents led to the complete consumption of HFC-125 and a TFE yield of 97% (entry 3). This condition, using 3.0 equivalents of KHMDS relative to the outflow of HFC-125, also allowed for an extension of the bubbling time to 30 min (HFC-125: 4.50 mmol), achieving a nearly quantitative transformation to TFE (>99% yield) (entry 4). Finally, when the amount of KHMDS used in entry 1 was reduced to 3.0 equivalents and the bubbling was repeated for 5 min, TFE was obtained in 67% yield (entry 5). Thus, under the optimal conditions (>99% yield, 4.5 mmol of TFE, entry 4), scaling up within the current laboratory setup would enable increased production, yielding approximately 9 mmol (900 mg) of TFE in 1 h and 90 mmol (9 g) over 10 h. Furthermore, even larger production volumes could be achieved by utilizing a larger-scale continuous flow system.

**Table 2:** Transformation of HFC-125 to TFE by a continuous flow process


Entry	Flow time (min)	Total HFC-125 (x mmol)	KHMDS (y equiv)	Yield (%)^a^	
				HFC-125	TFE
1	5	0.75	4.0	0	77
2	10	1.50	2.0	35	40
3	10	1.50	3.0	0	97
4	30	4.50	3.0	0	>99
5	5	0.75	3.0	0	67

a Yield was determined by^19^F NMR using C_6_F_6_ as the internal standard.

Our findings highlight that the stability and decomposition behavior of CF_3_CF_2_^−^ are fundamentally governed by the steric and electronic properties of strong bases (e.g., KHMDS) and the solvent environment (ether vs. non-ether systems). Notably, the microflow process provides a distinct advantage, enabling highly selective and controlled decomposition to TFE at room temperature. This offers a practical and scalable approach for the conversion of HFC-125. By advancing the mechanistic understanding of CF_3_CF_2_^−^ reactivity, this work contributes to the broader field of fluorine chemistry and supports the development of sustainable strategies for upcycling and repurposing HFCs in alignment with global environmental initiatives.

### Conclusions

We have developed a highly efficient and scalable process for the conversion of HFC-125, a greenhouse gas, to TFE, an essential monomer for the fluoropolymer industry. Our method, using KHMDS at low temperatures, not only achieves high TFE yields but also effective of repurposing HFC-125. Meanwhile, fluoride is recovered to form KF. Moreover, the continuous flow conversion of HFC-125 to TFE was successfully achieved at ambient temperature without the need for cryogenic conditions during the reaction. However, low-temperature liquefaction was required for the efficient collection of gaseous TFE. Furthermore, we demonstrated the broad utility of this process, successfully applying TFE in a range of chemical reactions, including thiol addition, trifluorovinylation, radical addition, amination, alcohol addition and radical polymerization for TFE polymer, each yielding significant products with practical applications. Beyond its impact on the fluoropolymer industry, this straightforward process for generating TFE offers significant potential for medicinal chemistry. The ease of TFE production could inspire its use in late-stage fluoro-functionalization of drug derivatives containing SH, OH, or NH groups, a crucial advancement given the growing importance of fluoroorganic compounds in both the pharmaceutical^92^ and agrochemical sectors.^93^

The conventional production of TFE relies on the high-temperature pyrolysis of R-22, a process that requires significant energy input for both heating and cooling. In contrast, our method enables the conversion of HFC-125 to TFE under ambient conditions using a continuous flow system, presenting a potentially more energy-efficient and sustainable alternative. If successfully scaled up, this approach could not only reduce the energy burden associated with TFE production but also provide an environmentally responsible strategy for repurposing HFC-125—a hydrofluorocarbon subject to global regulatory phase-down. By addressing both the environmental challenges of HFC-125 and the increasing demand for TFE, this work contributes meaningfully to fluorine chemistry, building upon the milestone discovery of the “taming of fluoroform (HFC-23)^94^” in 2012. Moreover, the dehydrofluorination strategy employed here could be extended to other HFCs, enabling the synthesis of a broader range of fluoroolefins, which are essential intermediates in organofluorine chemistry. With the ongoing global efforts to phase down HFC emissions under the Kigali Amendment to the Montreal Protocol, our approach provides a sustainable and economically viable alternative to conventional disposal methods. Instead of incinerating HFCs and generating CO_2_ emissions, this upcycling strategy offers a pathway to convert stocked HFCs into valuable fluorochemicals, supporting a circular and environmentally conscious fluoropolymer industry.

### Limitations of the study

While our method demonstrates broad applicability, from batch reactions to continuous flow processes, one current limitation lies in the collection and handling of the generated TFE. In industrial practice, TFE is typically stored and utilized as a compressed gas in high-pressure cylinders, which allows for precise pressure control during the subsequent fluoropolymer synthesis, such as the production of PTFE. In contrast, in our laboratory-scale setup, TFE is collected as a liquefied gas at low temperatures, which, while effective for small-scale chemical transformations, may limit its direct use in pressure-controlled polymerization processes. Therefore, establishing a safe and efficient method for collecting the generated TFE directly into a pressurized gas cylinder is a critical next step to fully realize the potential of this approach for sustainable fluoropolymer production at scale.

## Resource availability

### Lead contact

Further information and requests for resources and reagents should be directed to and will be fulfilled by the lead contact, Norio Shibata (nozshiba@nitech.ac.jp).

### Materials availability

This study did not generate new unique reagents.

### Data and code availability

• All data supporting the results reported in this study are available within this paper and the supplemental information or from the lead contact upon request.
• This paper does not report original code.
• Any additional information required to reanalyze the data reported in this paper is available from the lead contact upon request.

## Acknowledgments

This study was supported by the 10.13039/501100003382CREST program of the 10.13039/501100002241Japan Science and Technology Agency, entitled “Precise Material Science for Degradation and Stability” (grant number: JPMJCR21L1), and by Dr. Seiji Motojima (CMC Research Institute, Japan). J.E. thanks Universitat de València for financial support though the Fondo de investigación universitaria (FIU). J.E. also thanks the Advanced Materials programme (MFA/2022/051) supported by MCIN with funding from European Union NextGenerationEU (PRTR-C17.I1). The computational resources from the Servei d'Informàtica de la Universitat de València (SIUV) are gratefully acknowledged for providing access to supercomputing resources. We thank Mr. Yusuke Murata (Nagoya Institute of Technology) for his help with the reaction in Figure 4C.

## Author contributions

Conceptualization, N.S.; methodology, H.I., N.H., Y.K., and N.S.; investigation, H.I.; analyses, H.I. and N.H.; DFT calculations, J.E.; discussion, H.I., N.H., Y.K., J.E., and N.S.; funding acquisition, N.S.; project administration, N.S.; supervision, N.S.; writing – original draft, H.I. and N.S.; writing – review and editing, N.S.

## Declaration of interests

A patent application has been filed related to the research described in this manuscript.

## Declaration of generative AI and AI-assisted technologies

During the preparation of this work, N.S. used Paperpal (editage) and DeepL in order to check the accuracy of the usage of the English language and the correction of the grammar. After using this tool or service, N.S. reviewed and edited the content as needed and takes full responsibility for the content of the publication.

## STAR★Methods

### Key resources table

**Table:** 

REAGENT or RESOURCE	SOURCE	IDENTIFIER
**Chemicals, peptides, and recombinant proteins**		

Pentafluoromethane	Takachiho Chemical Industrial Co., Ltd.	CAS: 354-33-6
Potassium bis(trimethylsilyl)amide	Sigma-Aldrich	CAS: 40949-94-8 Cat# 324671
4-*tert*-Butylbenzenethiol	TCI	CAS: 2396-68-1 Cat# B0741
2-Naphthalenethiol	TCI	CAS: 91-60-1 Cat# N0020
Benzylmagnesium Chloride	Sigma-Aldrich	CAS: 6921-34-2 Cat# 225916
Phenylmagnesium Bromide	Sigma-Aldrich	CAS: 100-58-3 Cat# 171565
Lithium Chloride	Sigma-Aldrich	CAS: 7447-41-8 Cat# 203637
2,2′-Azobis(2,4-dimethylvaleronitrile)	TCI	CAS: 4419-11-8 Cat# A0680
2-(chlorotetrafluoro-l6-sulfaneyl)pyridine (**5a**)	Synthetized in our lab (Shibata et al.^79^)	https://doi.org/10.1002/anie.201605008
5-bromo-2-(chlorotetrafluoro-l6-sulfaneyl)pyridine (**5b**)	Synthetized in our lab (Shibata et al.^79^)	https://doi.org/10.1002/anie.201605008
Dimethylamine	TCI	CAS: 124-40-3 Cat# D3948
Piperidine	KANTO CHEMICAL CO., INC	CAS: 110-89-4 Cat# 32249-00
Sodium Hydrogen Carbonate	NACALAI TESQUE, INC.	CAS: 144-55-8 Cat# 31212-12
Benzyl Alcohol	TCI	CAS: 100-51-6 Cat# B2378
1-Hexanol	NACALAI TESQUE, INC.	CAS: 111-27-3 Cat# 18013-45
Potassium Hydroxide	NACALAI TESQUE, INC.	CAS: 1310-58-3 Cat# 28616-45
Diethylene Glycol Dimethyl Ether	TCI	CAS: 111-96-6 Cat# B0498
*tert*-butyl peroxypivalate	NOF CORPORATION	CAS: 927-07-1
Solkane®365mfc	NIPPON SOLVAY K.K.	CAS: 406-58-6

**Software and algorithms**		

Gaussian16	Frisch et al.^95^	RRID:SCR_014897; https://gaussian.com/
CYLview20	Legault, C. Y.^96^	http://www.cylview.org

### Experimental model and study participant details

This study did not use experimental models typical in life sciences.

### Method details

#### General information

##### Chemicals

Commercially available chemicals were obtained from Aldrich Chemical Co., Nacalai tesque, TCI, Wako and used as received unless otherwise stated. HFC-125 was purchased from Takachiho Chemical Industrial Co., Ltd. and used. The pure gaseous TFE supplied by Daikin Industries, Ltd. was used. *tert*-butyl peroxypivalate was given by NOF CORPORATION.

##### Solvents

All solvents were dehydrated and degassed and transferred with a syringe.

##### Column chromatography

Column chromatography was performed on silica gel 60N spherical neutral size, 64−210 μm (FUJIFILM Wako).

##### Nuclear magnetic resonance spectroscopy

NMR spectra were recorded on a Varian Mercury 300 spectrometer for ^1^H NMR (300 MHz) and ^19^F NMR (282 MHz), and a Bruker Avance 500 for ^1^H NMR (500 MHz) and ^13^C{^1^H} NMR (125 MHz) and a JEOL RESONANCE ECZ700R for ^1^H NMR (700 MHz). In ^19^F NMR experiments, Magritek Spinsolve60 (60 MHz) was used. Solid-state NMR was measured using JEOL JNM-ECA600 (600 MHz). The chemical shifts (δ) were measured in parts per million with respect to solvent (^1^H: TMS, δ = 0.00 ppm, ^19^F: CDCl_3_, δ = −162.2 ppm with C_6_F_6_ as internal standard; ^13^C{^1^H}: CDCl_3_, δ = 77.16 ppm), and coupling constants (*J*) are given in hertz. The following abbreviations denoted the corresponding multiplicities: s, singlet; d, doublet; t, triplet; q, quadruplet; dd, doublet of doublets; td, triplet of doublets; dt, doublet of triplets; m, multiplet; br, broad.

##### Mass spectrometry

Mass spectra were recorded using a JEOL JMS-Q1050GC (EI−GCMS) system and LCMS-2020EV (ESI-MS) system. High−resolution mass spectrometry (HRMS) was performed on a Waters Synapt G2 HDMS (ESI−TOF−MS) instrument.

##### Infrared absorption spectrometry

Infrared spectra were recorded using a JASCO FT/IR−6300 spectrometer.

##### Melting points

Melting points were determined using a BUCHI M−565 apparatus.

##### Cooling and heating source

A Techno Sigma UC Reactor was used as a cooling source. An oil bath was used as a heating source.

##### Reaction

All reactions were performed under an inert gas atmosphere unless otherwise noted.

##### Components of flow system

A mass flow controller (Fujikin, FCST1005MLC) was used to control the flow rate of gaseous HFC-125. The pressure of HFC-125 was regulated by the regulator connected to the HFC-125 cylinder. All flow apparatus (grass vials, stir bar, rubber septum, tubes) were dried and filled with N_2_ gas prior to use. A ETFE tube (inner diameter: 1.0 mm) was used for the gas flow path.

#### Experimental procedure and characterization

##### Procedure for determination of the yield of tetrafluoroethylene

In the glove box, a solution of toluene in which KHMDS was dissolved to make 0.25 M was prepared and a defined amount was transferred to an NMR tube. Toluene-d 8 was added to make a total volume of 0.7 mL, followed by 1.14 μL (0.01 mmol) of hexafluorobenzene as an internal standard. The NMR tube was covered with a septum cap, sealed with parafilm, and removed from glove box. The mixture was frozen in liquid nitrogen and vacuumed. HFC−125 was removed from the gas cylinder into a balloon and 0.56 mL (0.025 mmol) was injected under liquid nitrogen using a gastight syringe. While the lower part of the tube was immersed in liquid nitrogen, the upper part of the tube was warmed by hand to melt HFC−125 down to the solution interface. The mixture was moved to a low temperature methanol bath at −50°C and stirred vigorously for 3 h. The sample was moved to a low temperature methanol bath at −90°C and allowed to stand for 1 h, after which ^19^F NMR was measured while maintaining the temperature as low as possible to obtain the integrated value.

##### General procedure 1: Generation of tetrafluoroethylene

In the glove box, KHMDS (1.5 equiv) and stirrer tip were placed in a vacuum sample tube (chamber A) and dissolved in 3.0 mL of toluene. The tube was closed with a Teflon screw cap, a septum cap attached to the side arm, removed from the glow box and immersed in liquid nitrogen to freeze the solution. The inside of the container was vacuumed, and a predetermined amount of HFC−125 (1.0 equiv) was injected from the side tube side using a gas tight syringe (Figure S7). The mixture was moved to a low temperature bath at −50°C and stirred vigorously for 3 h.

##### General procedure 2: Hydrothiolation of TFE using thiol

**Figure:**
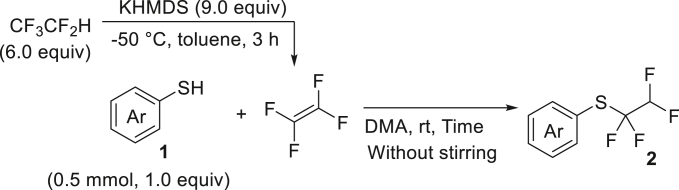

TFE was synthesized according to general procedure 1. Thiol **1** (0.5 mmol, 1.0 equiv), 12.3 μL PhCF_3_ (0.1 mmol) as an internal standard and stirrer tip were added to another oven−dried vacuum sample tube (chamber B) under a nitrogen atmosphere, dissolved in 3.0 mL of dry N,N-Dimethylacetamide (DMA), and sealed with a Teflon screw cap. The solution was frozen by immersion in liquid nitrogen, and the interior was decompressed. The side arms of chambers A and B were connected with a silicon tube, and the cap was opened slightly while the chamber B was immersed in liquid nitrogen (Figure S8). Then, the cap of the chamber A was opened slowly, and the TFE was transferred. Once the transfer was complete, all caps were closed and the silicone tube removed and the top of chamber B warmed to drop the TFE to the solution interface. The mixture was warmed up to room temperature and left to rest for 3 h. The reaction mixture was poured into 30 mL of water and extracted with Et_2_O (5 mL × 3). The combined organic layers were washed with Brine and dried over Na_2_SO_4_. The solvent was removed under reduced pressure with a rotary evaporator and the residue was purified by flash silica gel column chromatography to give compound **2**.

##### Scale-up synthesis of **2a** using autoclave

In the glove box, 18 mmol KHMDS (9.0 equiv), stirrer tip and 6.0 mL of toluene were added to a flame dried autoclave vessel and stirred vigorously. The autoclave was covered in a glove box before being removed and immersed in liquid nitrogen to freeze the solution. 1440 mg HFC−125 (12 mmol, 6.0 equiv) was injected while cooling with liquid nitrogen by reducing the internal pressure with a vacuum pump. The mixture was moved to a methanol bath at −50°C and stirred vigorously for 3 h. Thiol **1a** (334.8 mg, 2.0 mmol), stirrer tip, hexafluorobenzene 115 μL (1.0 mmol) as an internal standard and 12 mL dry DMA were added to a flame − dried autoclave with bubbling lines under argon atmosphere. It was tightly closed and cooled to −85°C. The autoclave which produced the TFE was removed from the cold bath and connected to the bubbling line. The pressure in the autoclave at the transfer destination was quickly reduced (Figure S9A). The line cock was slowly opened and TFE was slowly bubbled at −85°C over 1 h (Figure S9B). After the transfer was completed, the mixture was warmed to room temperature and left to rest for 3 h. The cock was opened to purge excess TFE in the draft. The reaction mixture was poured into 50 mL of water and extracted with Et_2_O (10 mL × 3). The combined organic layers were washed with Brine and dried over Na_2_SO_4_. The solvent was removed under reduced pressure with a rotary evaporator and the residue was purified by flash silica gel column chromatography (^*n*^Hexane) to give compound **2a** (517.3 mg, 97%).

##### (4-(tert-butyl)phenyl)(1,1,2,2-tetrafluoroethyl)sulfane (**2a**)

**Figure:**
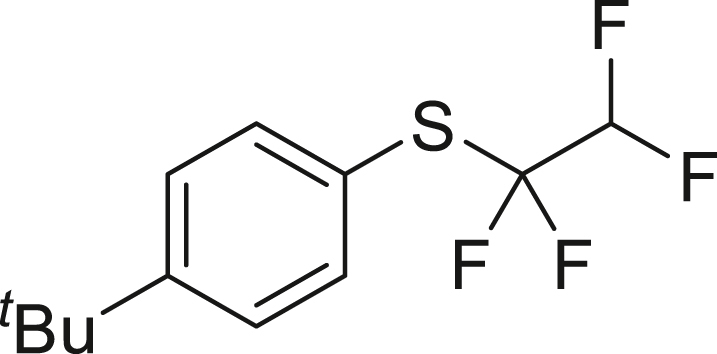

HFC-125(3.0 mmol, 67. 2 mL) and KHMDS (4.5 mmol, 897.7 mg) were transformed to TFE following general procedure 1. **2a** was obtained using **1a** (82.4 mg, 0.5 mmol) by following general procedure 2. Purification by column chromatography on silica gel (^*n*^hexane) to give **2a** (119.2 mg, 90%) as a colorless oil.

^**1**^**H NMR** (300 MHz, chloroform-d) δ: 7.57 (d, *J* = 8.5 Hz, 2H), 7.42 (d, *J* = 8.5 Hz, 2H), 5.76 (tt, ^1^*J* = 53.7 Hz, ^2^*J* = 3.6 Hz, 1H), 1.33 (s, 9H) ppm. ^**19**^**F NMR** (282 MHz, chloroform-d) δ: −93.0 (td, ^1^*J* = 9.7 Hz, ^2^*J* = 3.3 Hz, 2F), −133.5 (dt, ^1^*J* = 53.8 Hz, ^2^*J* = 9.4 Hz, 2F) ppm. **MS** (EI) *m*/*z*: [M]^+^ 266.0. Analytical data are consistent with reported values.^77^

##### naphthalen-2-yl(1,1,2,2-tetrafluoroethyl)sulfane (**2b**)

**Figure:**
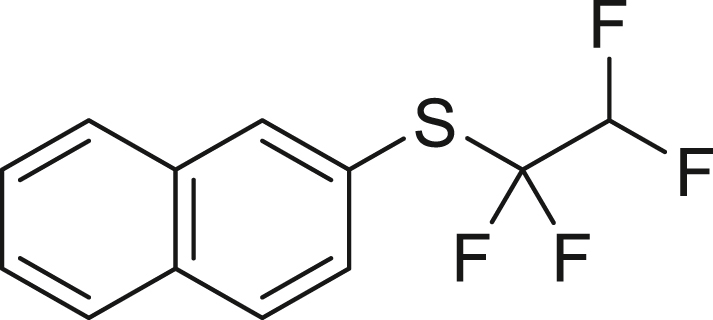

HFC-125(67.2 mL, 3.0 mmol) and KHMDS (897.7 mg, 4.5 mmol) were transformed to TFE following general procedure 1. **2b** was obtained using **1b** (79.7 mg, 0.5 mmol) by following general procedure 2. Purification by column chromatography on silica gel (^*n*^hexane) to give **2b** (105.4 mg, 81%) as a colorless oil.

^**1**^**H NMR** (300 MHz, chloroform-d) δ: 8.21 (s, 1H), 7.87 (d, *J* = 8.6 Hz, 3H), 7.67-7.54 (m, 3H), 5.80 (tt, ^1^*J* = 53.8 Hz, ^2^*J* = 3.4 Hz, 1H) ppm. ^**19**^**F NMR** (282 MHz, chloroform-d) δ: −92.2 (td, ^1^*J* = 9.4 Hz, ^2^*J* = 3.3 Hz, 2F), −133.5 133.0 (dt, ^1^*J* = 53.8 Hz, ^2^*J* = 9.2 Hz, 2F) ppm. **MS** (EI) *m*/*z*: [M]^+^ 260.0. Analytical data are consistent with reported values.^77^

##### General procedure 3: Trifluoro-vinylation with Grignard reagent

**Figure:**
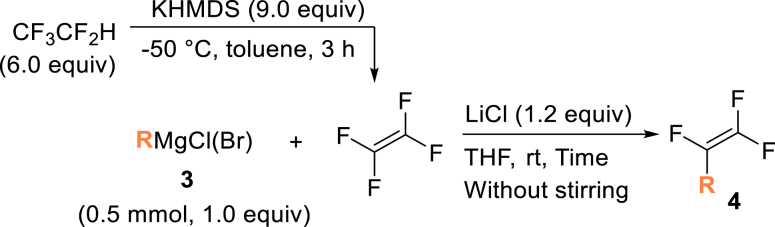

TFE was synthesized according to general procedure 1. In the glove box, lithium chloride (0.6 mmol, 1.2 equiv), Grignard reagent **3** (0.5 mmol, 1.0 equiv), 12.3 μL PhCF_3_ (0.1 mmol) as an internal standard and stirrer tip were added to another oven−dried vacuum sample tube (chamber B) and dissolved in 3.0 mL of dry THF and stirred at room temperature. It was sealed with a Teflon screw cap and removed from glove box. The solution was frozen by immersion in liquid nitrogen, and the interior was decompressed. The side arms of chambers A and B were connected with a silicon tube, and the cap was opened slightly while the chamber B was immersed in liquid nitrogen. Then, the cap of the chamber A was opened slowly, and the TFE was transferred. Once the transfer was complete, all caps were closed and the silicone tube removed and the top of chamber B warmed to drop the TFE to the solution interface. The temperature was raised to room temperature and left to rest for a predetermined time. The yield of compound **4** was determined by ^19^F NMR.

##### (2,3,3-trifluoroallyl)benzene (**4a**)

**Figure:**
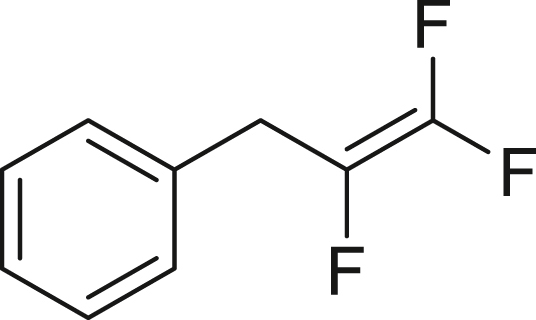

HFC-125(67.2 mL, 3.0 mmol) and KHMDS (897.7 mg, 4.5 mmol) were transformed to TFE following general procedure 1. Following general procedure 3, benzyl magnesium chloride (**3a**; in 2.0 M THF solution, 0.25 mL, 0.5 mmol) and LiCl (25.4 mg, 0.6 mmol) gave the title compound in >99% yield by placing still without stirring for 3 h. ^**19**^**F NMR** (282 MHz, chloroform-d) δ: −105.6 (dd, ^1^*J* = 85.7 Hz, ^2^*J* = 32.2 Hz, 1F), −124.5 (dd, ^1^*J* = 114.6 Hz, ^2^*J* = 85.9 Hz, 1F), −172.9 (ddt, ^1^*J* = 114.8 Hz, ^2^*J* = 41.4, ^3^*J* = 16.1 Hz, 1F). **MS (EI)** m/z: [M]^+^ 172.0. Analytical data are consistent with reported values.^78^

##### (1,2,2−trifluorovinyl)benzene (**4b**)

**Figure:**
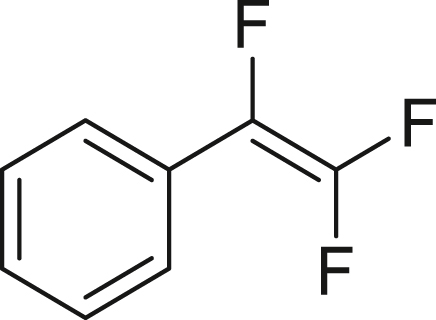

HFC-125(112.0 mL, 5.0 mmol) and KHMDS (1496.1 mg, 7.5 mmol) were transformed to TFE in 4.0 mL of toluene following general procedure 1. Following general procedure 3, phenyl magnesium bromide (**3b**; in 3.0 M Diethyl ether solution, 0.2 mL, 0.6 mmol) and LiCl (30.5 mg, 0.72 mmol) gave the title compound in 74% yield by placing still without stirring for 3 days ^**19**^**F NMR** (282 MHz, chloroform-d) δ: −100.3 (dd, ^1^*J* = 71.3 Hz, ^2^*J* = 32.7 Hz, 1F), −115.1 (dd, ^1^*J* = 110.0 Hz, ^2^*J* = 71.3 Hz, 1F), −177.2 (dd, ^1^*J* = 109.0 Hz, ^2^*J* = 32.7 Hz, 1F). **MS (EI)** m/z: [M]^+^ 158.0. Analytical data are consistent with reported values.^97^

##### General procedure 4: Radical addition of PySF_4_Cl

**Figure:**
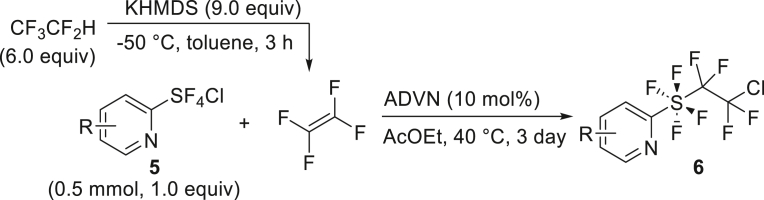

TFE was synthesized according to general procedure 1. In the glove box, PySF_4_Cl **5** (0.5 mmol, 1.0 equiv), stirrer tip and ADVN (0.05 mmol, 10 mol%) were added to another oven−dried vacuum sample tube (chamber B) and dissolved in 2.0 mL of dry ethyl acetate. It was sealed with a Teflon screw cap and removed from glove box. The solution was frozen by immersion in liquid nitrogen, and the interior was decompressed. The side arms of chambers A and B were connected with a silicon tube, and the cap was opened slightly while the chamber B was immersed in liquid nitrogen. Then, the cap of the chamber A was opened slowly, and the TFE was transferred. Once the transfer was complete, all caps were closed and the silicone tube removed and the top of chamber B warmed to drop the TFE to the solution interface. The mixture was warmed up to 40°C and stirred at same temperature for 3 days. The solvent was removed under reduced pressure with a rotary evaporator and the residue was purified by flash silica gel column chromatography to give compound **6**.

##### 2-((2-chloro-1,1,2,2-tetrafluoroethyl)tetrafluoro-λ^6^-sulfaneyl)pyridine (**6a**)

**Figure:**
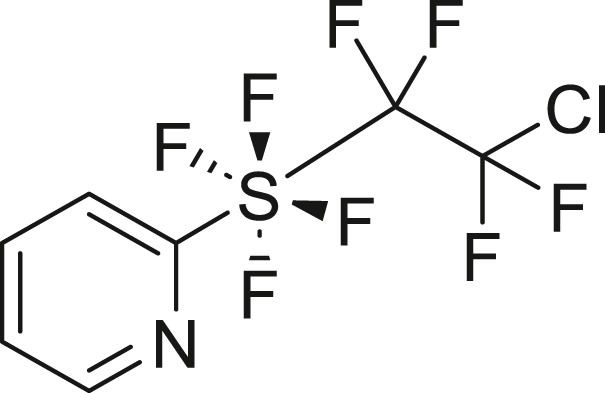

HFC-125(67.2 mL, 3.0 mmol) and KHMDS (897.7 mg, 4.5 mmol) were transformed to TFE following general procedure 1. **6a** was obtained using **5a** (117.1 mg, 0.5 mmol) and ADVN (12.4 mg, 0.05 mmol) by following general procedure 4. Purification by silica gel column chromatography (^*n*^hexane/AcOEt = 20/1) to give **6a** (85.9 mg, 51%) as a colorless oil. ^**1**^**H NMR** (300 MHz, CDCl_3_) δ: 8.60 (d, *J* = 3.2 Hz, 1H), 7.94 (t, *J* = 7.9 Hz, 1H), 7.78 (d, *J* = 8.2 Hz, 1H), 7.51 (dd, ^1^*J* = 7.3 Hz, ^2^*J* = 4.7 Hz, 1H) ppm ^**19**^**F NMR** (282 MHz, CDCl_3_) δ: 37.5–37.3 (m, 4F), −67.9–−68.1 (m, 2F), −91.1–−91.3 (m, 2F) ppm. **MS (EI)** m/z: [M + Na]^+^ 321.0. Analytical data are consistent with reported values.^82^

##### 5-bromo-2-((2-chloro-1,1,2,2-tetrafluoroethyl)tetrafluoro-λ^6^-sulfaneyl)pyridine (**6b**)

**Figure:**
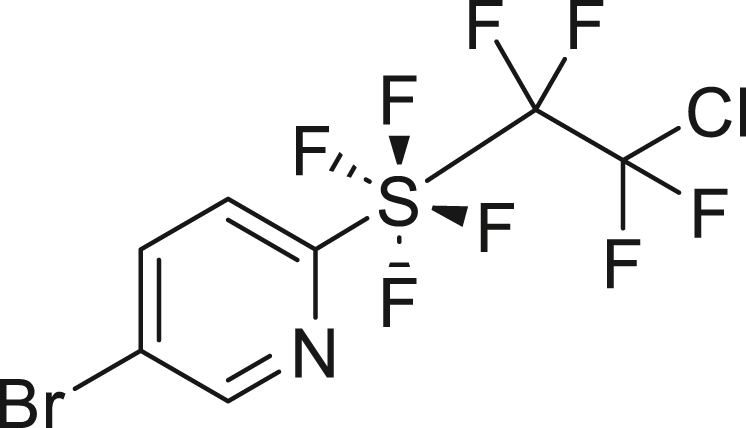

HFC-125(67.2 mL, 3.0 mmol) and KHMDS (897.7 mg, 4.5 mmol) were transformed to TFE following general procedure 1. **6b** was obtained using **5b** (180.3 mg, 0.6 mmol) and ADVN (14.9 mg, 0.06 mmol) by following general procedure 4. Purification by silica gel column chromatography (^*n*^hexane/AcOEt = 20/1) to give **6b** (179.6 mg, 76%) as a pale-blown solid. ^**1**^**H NMR** (300 MHz, CDCl_3_) δ: 8.63 (d, *J* = 2.1 Hz, 1H), 8.05 (d, *J* = 8.5 Hz, 1H), 7.68 (d, *J* = 8.8 Hz, 1H) ppm ^**13**^**C {**^**1**^**H} NMR** (126 MHz, CDCl_3_) δ: 165.0 (quint, *J* = 25.2 Hz), 149.1, 141.4, 124.1, 122.8 (t, *J* = 4.1 Hz), 122.2 (tt, ^1^*J* = 303.8 Hz, ^2^*J* = 35.4 Hz), 121.0 (tquint, ^1^*J* = 321.3 Hz, ^2^*J* = 37.3 Hz) ppm ^**19**^**F NMR** (282 MHz, CDCl_3_) δ: 38.6–38.4 (m, 4F), −68.1–−68.2 (m, 2F), −91.1–−91.3 (m, 2F) ppm. FT-IR (KBr): ν = 3056, 2924, 1567, 1451, 1366, 1210, 1138, 800, 664 cm^-1^. **m.p.**: 50.3°C–51.3°C. **HRMS (ESI)** m/z: [M + Na]^+^ calcd. for C_7_H_3_ClF_8_NSNa; 421.8628 Found 421.8629.

##### General procedure 5: Addition reaction of amines using TFE

**Figure:**
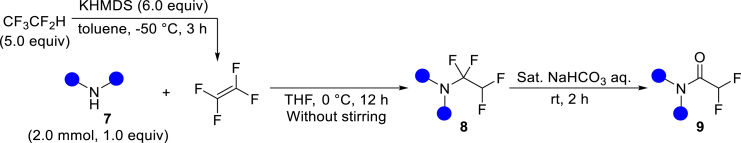

In the glove box, 12 mmol KHMDS (6.0 equiv) and stirrer tip and 6.0 mL of toluene were added to a flame dried autoclave vessel and stirred vigorously. The autoclave was covered in a glove box before being removed and immersed in liquid nitrogen to freeze the solution. 1200 mg HFC−125 (10 mmol, 5.0 equiv) was injected while cooling with liquid nitrogen by reducing the internal pressure with a vacuum pump. The mixture was moved to a methanol bath at −50°C and stirred vigorously for 3 h. Amine **7** (2.0 mmol, 1.0 equiv), hexafluorobenzene 115 μL (1.0 mmol) as an internal standard and 5 mL THF were added to a flame−dried autoclave with bubbling lines under argon atmosphere. It was tightly closed and cooled to −94°C. The autoclave which produced the TFE was removed from the cold bath and connected to the bubbling line. The pressure in the autoclave at the transfer destination was quickly reduced. The line cock was slowly opened and TFE was slowly bubbled at −94°C over 1 h. After the transfer was completed, the temperature of the bath was slowly raised to 0°C over 3 h and placed still without stirring at 0°C for 12 h. After the mixture was warmed up to room temperature, the cock was opened to purge excess TFE in the draft. The yield of compound **8** was determined by ^19^F NMR. The saturated aqueous NaHCO_3_ (10 mL) was added dropwise to the mixture at 0°C. and stirred at room temperature for 2 h. The aqueous layer was extracted with Et_2_O, and the combined organic layers were washed with brine, dried over Na_2_SO_4_. The organic layer was concentrated under reduced pressure and dried in vacuo. The amide compound **9** was obtained without further purification.

##### 1,1,2,2–tetrafluoro–*N*, *N*–dimethylethan–1–amine (**8a**)

**Figure:**
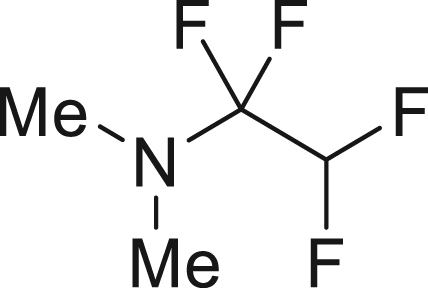

Following the general procedure 5, Dimethylamin (**7a**; in 2.0 M THF solution, 1.0 mL, 2.0 mmol, 1.0 equiv) gave the title compound in >99% yield.

^**19**^**F NMR** (282 MHz, chloroform-d) δ −102.5 (s, 2F), −134.0 (dt, ^1^*J* = 53.2 Hz, ^2^*J* = 6.2 Hz, 2F) ppm. **MS** (EI), *m*/*z*: [M]^+^ 145.0. Analytical data are consistent with reported values.^83^

##### 1-(1,1,2,2-tetrafluoroethyl)piperidine (**8b**)

**Figure:**
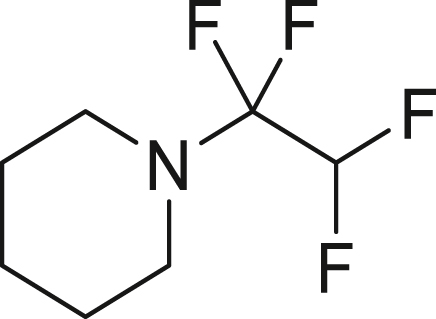

Following the general procedure 5, piperidine **7b** (170.3 mg, 2.0 mmol, 1.0 equiv) gave the title compound in 88% yield.

^**19**^**F NMR** (282 MHz, chloroform-d) δ −100.4 (s, 2F), −134.0 (dt, ^1^*J* = 53.2 Hz, ^2^*J* = 6.4 Hz, 2F) ppm. Analytical data are consistent with reported values.^98^

##### 2,2-difluoro-*N*, *N*-dimethylacetamide (**9a**)

**Figure:**
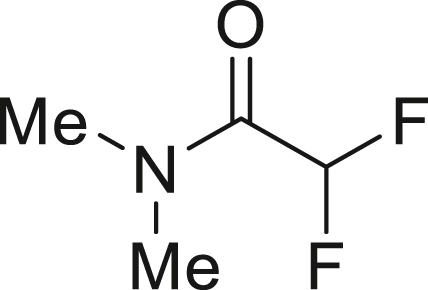

Treatment of a mixture of **8a** with sat. NaHCO_3_ aq. according to general procedure 5 gave **9a** (198.0 mg, 80%) as a yellow oil.

^**1**^**H NMR** (700 MHz, chloroform-d) δ: 6.11 (t, *J* = 53.7 Hz, 1H), 3.14 (t, *J* = 1.4 Hz, 3H), 3.01 (t, *J* = 1.0 Hz, 3H) ppm ^**19**^**F NMR** (282 MHz, chloroform-d) δ: −122.2 (d, *J* = 54.1 Hz, 2F) ppm. **MS** (EI) *m*/*z*: [M]^+^ 123.0. Analytical data are consistent with reported values.^99^

##### 2,2-difluoro-1-(piperidin-1-yl)ethan-1-one (**9b**)

**Figure:**
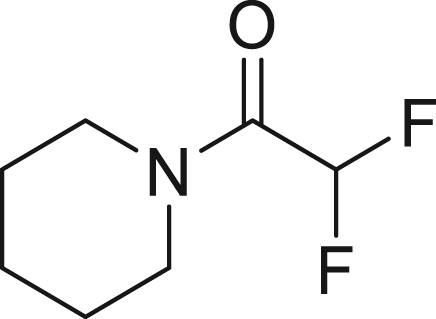

Treatment of a mixture of **8a** with sat. NaHCO_3_ aq. according to general procedure 5 gave **9b** (247.7 mg, 76%) as a yellow oil.

^**1**^**H NMR** (300 MHz, chloroform-d) δ: 6.11 (t, *J* = 53.8 Hz, 1H), 3.59-3.54 (m, 4H), 1.66-1.61 (m, 6H) ppm. ^**13**^**C {**^**1**^**H} NMR** (126 MHz, CDCl_3_) δ: 160.5 (t, *J* = 25.0 Hz), 110.6 (t, *J* = 253.8 Hz), 45.9 (t, *J* = 4.1 Hz), 43.7, 26.4, 25.4, 24.3 ppm. ^**19**^**F NMR** (282 MHz, chloroform-d) δ: −121.7 (d, *J* = 53.5 Hz, 2F) ppm. **FT-IR** (NaCl): ν = 2944, 2857, 1671, 1447, 1378, 1254, 1117, 1056, 856 cm^-1^. **HRMS (ESI)** m/z: [M + Na]^+^ calcd. for C_7_H_11_F_2_NONa; 186.0706 Found 186.0705.

##### General procedure 6: Addition reaction of alcohols using TFE

**Figure:**
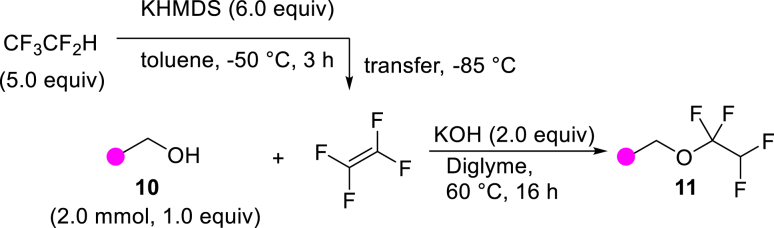

12 mmol KHMDS (6.0 equiv), stirrer tip and 6.0 mL of toluene were added to a flame dried autoclave vessel and stirred vigorously. The autoclave was covered in a glove box before being removed and immersed in liquid nitrogen to freeze the solution. 1200 mg HFC−125 (10 mmol, 5.0 equiv) was injected while cooling with liquid nitrogen by reducing the internal pressure with a vacuum pump. The mixture was moved to a methanol bath at −50°C and stirred vigorously for 3 h. Alcohol **10** (2.0 mmol, 1.0 equiv), stirrer tip, potassium hydroxide (4.0 mmol, 2.0 equiv), hexafluorobenzene 115 μL (1.0 mmol) as an internal standard and 10 mL dry Diglyme were added to a flame − dried autoclave with bubbling lines under argon atmosphere. It was tightly closed and cooled to −85°C. The autoclave which produced the TFE was removed from the cold bath and connected to the bubbling line. The pressure in the autoclave at the transfer destination was quickly reduced. The line cock was slowly opened and TFE was slowly bubbled at −85°C over 1 h. After the transfer was completed, the mixture was warmed to 60°C with oil bath and stirred slowly for 16 h. After the mixture was cooled to room temperature, the cock was opened to purge excess TFE in the draft. The mixture was quenched with aqueous ammonium chloride and extracted with 30 mL of water and Et_2_O (5 mL × 3). The combined organic layers were washed with Brine and dried over Na_2_SO_4_. The solvent was removed under reduced pressure with a rotary evaporator and the residue was purified by flash silica gel column chromatography to give the alcohol adduct **11**.

##### ((1,1,2,2-tetrafluoroethoxy) methyl) benzene (**11a**)

**Figure:**
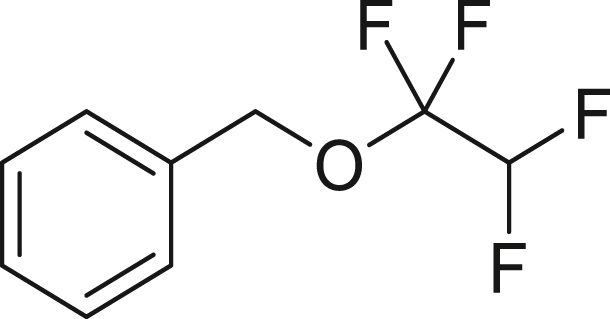

**11a** was obtained using **10a** (218.5 mg, 2.0 mmol) and KOH (224.4 mg, 4.0 mmol) by following general procedure 6. Purification by column chromatography on silica gel (^*n*^hexane/ethyl acetate = 98/2) to give **11a** (392.1 mg, 94%) as a colorless oil.

^**1**^**H NMR** (300 MHz, chloroform-d) δ: 7.42-7.38 (m, 5H), 5.75 (tt, ^1^*J* = 53.3 Hz, ^2^*J* = 2.9 Hz, 1H), 5.01 (s, 2H) ppm ^**19**^**F NMR** (282 MHz, chloroform-d) δ: −91.5 (td, ^1^*J* = 5.9 Hz, ^2^*J* = 2.9 Hz, 2F), −137.0 (dt, ^1^*J* = 53.5 Hz, ^2^*J* = 5.9 Hz, 2F) ppm. **MS** (EI) *m*/*z*: [M]^+^ 208.0. Analytical data are consistent with reported values.^100^

##### 1-(1,1,2,2-tetrafluoroethoxy)hexane (**11b**)

**Figure:**
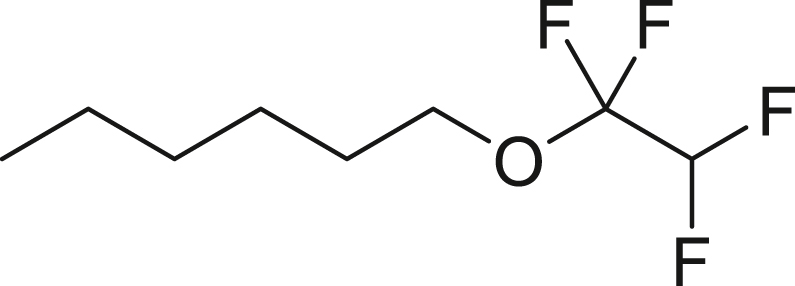

**11b** was obtained using **10b** (204.0 mg, 2.0 mmol) and KOH (224.4 mg, 4.0 mmol) by following general procedure 6. Purification by short open column chromatography on silica gel (^*n*^pentane) to give **11b** (216.1 mg, 54%) as a colorless oil.

^**1**^**H NMR** (300 MHz, chloroform-d) δ: 5.69 (tt, ^1^*J* = 53.4 Hz, ^2^*J* = 2.8 Hz, 1H), 3.97 (t, *J* = 6.6 Hz, 2H), 1.72-1.62 (m, 2H), 1.40-1.26 (m, 6H), 0.90 (t, *J* = 6.7 Hz, 3H) ppm. ^**13**^**C {**^**1**^**H} NMR** (126 MHz, CDCl_3_) δ: 117.5 (tt, ^1^*J* = 267.0 Hz, ^2^*J* = 28.2 Hz), 110.3-105.7 (m), 64.8-64.6 (m), 31.4, 29.0, 25.4, 22.6, 14.1 ppm ^**19**^**F NMR** (282 MHz, chloroform-d) δ: −91.9 (td, ^1^*J* = 5.9 Hz, ^2^*J* = 3.0 Hz, 2F), −137.2 (dt, ^1^*J* = 53.5, ^2^*J* = 5.9 Hz, 2F) ppm. **FT-IR** (NaCl): ν = 2961, 2932, 2862, 1277, 1219, 1124, 1084 cm^-1^. **LCMS (ESI)** m/z: [M + H]^+^ 203.10.

##### General procedure 7: Radical polymerization of the generated TFE

**Figure:**
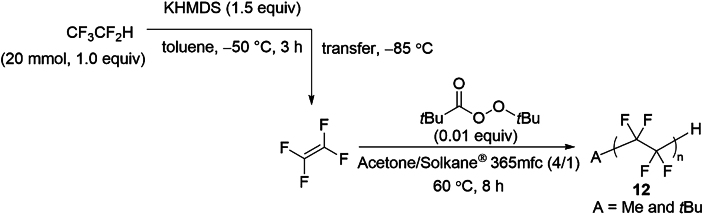

6 g KHMDS (30 mmol, 1.5 equiv), stirrer tip and 16.0 mL of toluene were added to a flame dried autoclave vessel and stirred vigorously. The autoclave was covered in a glove box before being removed and immersed in liquid nitrogen to freeze the solution. 2.4 g HFC−125 (20 mmol, 1.0 equiv) was injected while cooling with liquid nitrogen by reducing the internal pressure with a vacuum pump. The mixture was moved to a methanol bath at −50°C and stirred vigorously for 3 h. After 3 h, the mixture was removed from the methanol bath and allowed to warm to room temperature. A stirrer tip, 49.8 mg *tert*-butyl peroxypivalate (71% hydrocarbon dilution solution, 0.01 equiv), 12 mL degassed acetone and 3.0 mL Solkane-365mfc were added to a flame − dried another autoclave reactor with bubbling line at 0°C under nitrogen atmosphere. This mixture was bubbled with nitrogen at 0°C for 30 min. The reactor was evacuated and backfilled with nitrogen 4 times at 0°C. The reactor was evacuated again, and the autoclaves were connected. While the autoclave reactor for polymerization was cooled to −85°C, the valve was opened slowly to transfer the generated TFE. After the transfer was completed, the mixture was allowed to warm to 60°C with oil bath and stirred for 8 h. After the pressure stopped decreasing, the mixture was cooled to room temperature and de-pressurized to remove excess TFE in the draft. The solid product was filtered by vacuum and washed with acetone. The white solid was dried under vacuum to give the title compound **12**.

##### Polytetrafluoroethylene (**12**)

**Figure:**
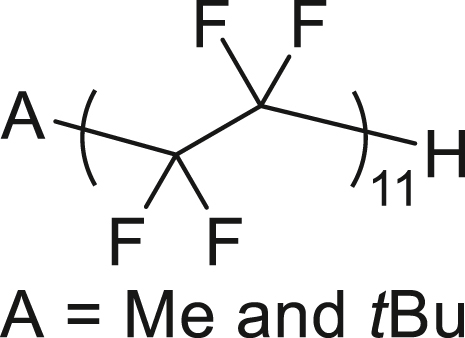

The title compound **12** (n = 11, 240 mg) was obtained as a white solid following general procedure 7.

^**1**^**H NMR** (600 MHz) δ: 6.00 (s, 1H), 1.36-1.08 (br) ppm. ^**13**^**C {**^**1**^**H} NMR** (151 MHz) δ: 111.5 ppm ^**19**^**F NMR** (565 MHz) δ: −112.7 (br, 2F), −122.1 (s, 38F), −130.3 (br, 2F), −138.3 (s, 2F) ppm.

##### General procedure for continuous flow synthesis of TFE

**Figure:**
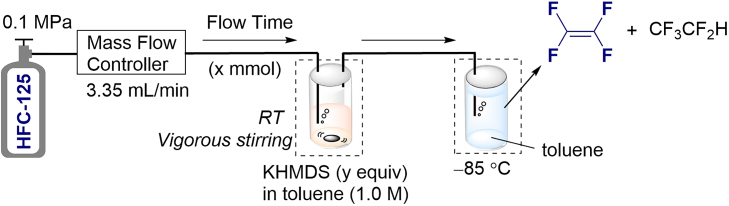

A 1.0 M KHMDS (3.0 equiv) -anhydrous toluene solution (reaction vessel) was prepared in a glove box oven-dried vial (5 min & 10 min: 10 mL, 30 min: 25 mL) based on the outflow time (5 min: 0.75 mmol, 10 min: 1.5 mmol, 30 min: 4.5 mmol) of HFC-125. A toluene solution (5min: 20 mL, 10 min: 23 mL, 30 min: 45 mL) with hexafluorobenzene (5 min: 0.1 mmol, 10 min & 30 min: 1.0 mmol) as an internal standard was prepared in a screw vial (5min & 10 min: 25 mL, 30 min: 50 mL) (collection vessel). The flow line as shown in Figure S10 was assembled under nitrogen atmosphere. At this time, each gas outlet was placed above the liquid surface, and the collection vessel was opened by inserting a needle. HFC-125 was flowed at a rate of 3.35 mL/min through a mass flow controller at a pressure of 0.1 MPa from a cylinder of HFC-125. The outlet of the HFC-125 outflow tube was immersed in the solution of the reaction vessel and bubbled. The reaction was started by vigorous stirring at room temperature. After exhausting the gas for 1 minute, the needle inserted into the collection vessel was removed to make a closed system. Immediately, the outlet of the outflow tube on the collection vessel side was immersed in toluene cooled to −85°C, and collection of TFE started. After the HFC-125 was continued to flow for a predetermined time (5 min or 10 min or 30 min), the flow of HFC-125 was stopped and the mixture was stirred at room temperature for another 5 minutes (Figure S11A). After no bubbles were generated, the tube tip of the collection vessel was removed from the toluene, and the collection vessel was immersed in liquid nitrogen to completely remove the gases in the reaction vessel (Figure S11B). After no bubbles were generated from the reactor, the collection vessel was closed tightly with a screw cap and immersed in a methanol bath at −85°C. After standing in a methanol bath at −85°C for 1 h, the mixture was quickly taken into an NMR tube previously cooled to −85°C, and ^19^F NMR was measured to determine the yield.

### Quantification and statistical analysis

#### Computational details

All density functional theory (DFT) calculations were carried out using the Gaussian16 package.^95^ All the structures were optimized using the long-range corrected hybrid ωB97xD density functional^101^ in combination with the Def2TZVP basis set.^102^ The effect of the solvent was mimicked by applying the SMD model using ether as solvent.^103^ The nature of the stationary points was confirmed by frequency calculations analysis at the same level of theory (minima were characterized by no imaginary frequencies, whereas transition states had one imaginary frequency). Transition states were further verified by relaxing the imaginary frequency towards the reactant and the product and by means of IRC calculations.^104^ 3D structures of optimized stationary points were represented using the CYLview 1.0 program.^96^

## Supplemental information

Document S1. Figures S1–S11, Tables S1 and S2, and Data S1 and S2

Data S3. Raw data of TFE calibration curve, related to Table 1

Data S4. Raw data of computational science, related to Figure 3

## References

[1] Wilson L.C., Wilding W.V., Wilson G.M., Rowley R.L., Felix V.M., Chisolm-Carter T. (1992). Thermophysical Properties of HFC-125. Fluid Phase Equilib..

[2] Yang Z., Liu H., Wu X. (2012). Theoretical and Experimental Study of the Inhibition and Inert Effect of HFC125, HFC227ea and HFC13I1 on the Flammability of HFC32. Process Saf. Environ. Prot..

[3] Nagy B., Csontos B., Csontos J., Szakács P., Kállay M. (2014). High-Accuracy Theoretical Thermochemistry of Fluoroethanes. J. Phys. Chem. A.

[4] Rusch G.M. (2018). The Development of Environmentally Acceptable Fluorocarbons. Crit. Rev. Toxicol..

[5] McCulloch A. (1999). CFC and Halon Replacements in the Environment. J. Fluor. Chem..

[6] Velders G.J.-M., Ravishankara A.R., Miller M.K., Molina M.J., Alcamo J., Daniel J.S., Fahey D.W., Montzka S.A., Reimann S. (2012). Preserving Montreal Protocol Climate Benefits by Limiting HFCs. Science.

[7] Flerlage H., Velders G.J.M., de Boer J. (2021). A review of Bottom-up and Top-down Emission Estimates of Hydrofluorocarbons (HFCs) in Different parts of the World. Chemosphere.

[8] Miranda N.D., P.-Alcantar P.G., Khosla R., McCulloch M.D. (2023). Metrics for the Emissions of F-gas Refrigerants. Sustain. Energy Technol Assess.

[9] Liu H., Duan H., Zhang N., Ma Y., Liu G., Miller T.R., Mao R., Xu M., Li J., Yang J. (2024). Rethinking Time-Lagged Emissions and Abatement Potential of Fluorocarbons in the Post-Kigali Amendment Era. Nat. Commun..

[10] U.S. Environmental Protection Agency Home Page (2024). Protecting Our Climate by Reducing Use of HFCs. https://www.epa.gov/climate-hfcs-reduction.

[11] Acerboni G., Beukes J.A., Jensen N.R., Hjorth J., Myhre G., Nielsen C.J., Sundet J.K. (2001). Atmospheric Degradation and Global Warming Potentials of Three Perfluoroalkenes. Atmos. Environ. X..

[12] Siegemund G., Schwertfeger W., Feiring A., Smart B., Behr F., Vogel H., McKusick B., Kirsch P. (2016). Ullmann's Encyclopedia of Industrial Chemistry.

[13] Downing F.B., Benning A.F., McHarness R.C. (1945). Pyrolysis of Chloro-fluoro Alkanes. U. S. Patent US2551573.

[14] Chinoy P.B., Sunavala P.D. (1987). Thermodynamics and Kinetics for the Manufacture of Tetrafluoroethylene by the Pyrolysis of Chlorodifluoromethane. Ind. Eng. Chem. Res..

[15] Farlow M.W. (1963). Method for the Preparation of Tetrafluoroethylene. U. S. Patent US3081245.

[16] Tress W.R.W. (1964). Preparation of Tetrafluoroethylene. U. S. Patent US3133871.

[17] Park J., Benning A., Downing F., Laucius J., McHarness R. (1947). Synthesis of Tetrafluorethylene-Pyrolisis of Monochlorodifluoromethane. Ind. Eng. Chem..

[18] Sung D.J., Moon D.J., Lee Y.J., Hong S.-I. (2004). Catalytic Pyrolysis of Difluorochloromethane to Produce Tetrafluoroethylene. Int. J. Chem. Reactor Eng..

[19] Yang G.-C., Jia X.-Q., Pan R.-M., Quan H.-D. (2009). The Disproportionation of CF_2_ Carbene in Vapor-Phase Pyrolysis Reaction over Activated Carbon and Porous Aluminum Fluoride. J. Mol. Catal. A: Chem..

[20] Timperley C.M. (2000). Chapter 29 - Highly-Toxic Fluorine Compounds. Fluorine Chemistry at the Millennium.

[21] Smith L.W., Gardner R.J., Kennedy G.L. (1982). Short-Term Inhalation Toxicity of Perfluoroisobutylene. Drug Chem. Toxicol..

[22] Wang H., Ding R., Ruan J., Yuan B., Sun X., Zhang X., Yu S., Qu W. (2001). Perfluoroisobutylene-Induced Acute Lung Injury and Mortality are Heralded by Neutrophil Sequestration and Accumulation. J. Occup. Health.

[23] Patocka J. (2019). Perfluoroisobutene: Poisonous Choking Gas. Mil. Med. Sci. Lett..

[24] Timperley C.M. (2004). Fluoroalkene Chemistry: Part 1. Highly-Toxic Fluorobutenes and their Mode of Toxicity: Reactions of Perfluoroisobutene and Polyfluorinated Cyclobutenes with Thiols. J. Fluor. Chem..

[25] Bauer G.L., Weigelt J.D., Hintzer K., Loehr G., Schwertfeger W., Ponelis A.A. (2004). Process for Manufacturing Fluoroolefins. U. S. Patent US20040112758.

[26] Zipplies T.C., Hintzer K., W.-Porada M.A., Gerdes T., Herdegen J., S.-Rodenkirchen A., Aschauer S. (2016). Process and Apparatus for Producing Fluorinated Alkenes. WO Patent WO2016054246.

[27] Ota T., Nakaya H., Hirai M., Yasuhara T., Noguchi A. (2019). Method for Producing at Least One of Tetrafluoroethylene and Hexafluoropropylene by Pyrolysis of Perfluorooctene and/or Perfluorodecylethylene. JP Patent JP2019199456.

[28] Benning A.F., Downing F.B., Plunkett R.J. (1946). Preparation of Tetrafluoroethylene. U. S. Patent US2401897.

[29] Mantell R.M. (1954). Dehalogenation of Fluorohalocarbons. U. S. Patent US2697124.

[30] Locke E.G., Brode W.R., Henne A.L. (1934). Fluorochloroethanes and Fluorochloroethylenes. J. Am. Chem. Soc..

[31] Farnham W.B. (1994). Process for the Production of Fluorinated Olefins. U. S. Patent US5347058.

[32] Hercules D.A., Parrish C.A., Sayler T.S., Tice K.T., Williams S.M., Lowery L.E., Brady M.E., Coward R.B., Murphy J.A., Hey T.A. (2017). Preparation of Tetrafluoroethylene from the Pyrolysis of Pentafluoropropionate Salts. J. Fluor. Chem..

[33] Lewis E.E., Naylor M.A. (1947). Pyrolysis of Polytetrafluoroethylene. J. Am. Chem. Soc..

[34] Waritz R.S. (1975). An Industrial Approach to Evaluation of Pyrolysis and Combustion Hazards. Environ. Health Perspect..

[35] Hunadi R., Baum K. (1982). Tetrafluoroethylene: A Convenient Laboratory Preparation. Synthesis.

[36] Simon C.M., Kaminsky W. (1998). Chemical Recycling of Polytetrafluoroethylene by Pyrolysis. Polym. Degrad. Stab..

[37] Puts G.J., Crouse P.L. (2014). The Influence of Inorganic Materials on the Pyrolysis of Polytetrafluoroethylene. Part 1: The Sulfates and Fluorides of Al, Zn, Cu, Ni, Co, Fe and Mn. J. Fluor. Chem..

[38] Puts G.J., Crouse P.L. (2014). The Influence of Inorganic Materials on the Pyrolysis of Polytetrafluoroethylene. Part 2: The Common Oxides of Al, Ga, In, Zn, Cu, Ni, Co, Fe, Mn, Cr, V, Zr and La. J. Fluor. Chem..

[39] Bezuidenhoudt A., Sonnendecker P.W., Crouse P.L. (2017). Temperature and Pressure Effects on the Product Distribution of PTFE Pyrolysis by Means of Qualitative, in-line FTIR Analysis. Polym. Degrad. Stab..

[40] Ellis D.A., Mabury S.A., Martin J.W., Muir D.C. (2001). Thermolysis of Fluoropolymers as a Potential Source of Halogenated Organic Acids in the Environment. Nature.

[41] Gelblum P.G., Herron N., Noelke C.J., Rao V.N.M. (2002). Synthesis of Perfluoroolefins. WO Patent WO2002006193.

[42] Hintzer K., Streiter A., Kaempf G.J., Lochhaas K.H., Juergens M., Shyshkov O., Zipplies T.C., Troe J., Luther K. (2012). Process for Manufacturing Perfluoroolefins by Pyrolysis of Perfluorocarbons in the Presence of Hydrogen. WO Patent WO2012012113.

[43] Ahmat X., Yoshino G., Lee H.D., Jung J.K. (2016). Method for Producing Tetrafluoroethylene and/or Hexafluoropropylene. JP Patent JP2016013994.

[44] Ding C., Wang X., Wang W., Du R., Han C., Sun S., Xu Q., Zhang X. (2017). Method for Preparing Hydrofluoroether with 1,1,1,2-Tetrafluoroethane. CN Patent CN107353184.

[45] Chen Q.Y., Qiu Z.M. (1986). Studies on Fluoroalkylation and Fluoroalkoxylation. Part 10. Electron-Transfer Induced Reactions of Perfluoroalkyl Iodides and the Dialkyl Malonate Anion and β-Fragmentation of the Halotetrafluoroethyl Radical. J. Fluor. Chem..

[46] Chen Q.-Y., Qiu Z.-M. (1987). Studies on Fluoroalkylation and Fluoroalkoxylation. Part 16. Reactions of Fluoroalkyl Iodides with Some Nucleophiles by S_RN_1 echanism. J. Fluor. Chem..

[47] Nakagawa S. (2015). Chain Reaction on De-halogenation of 1,2-Dibromotetrafluoroethane and 1,1,2-Trichlorotrifluoroethane Induced by Irradiation in Alcohols. Rad. Phys. Chem..

[48] Li L., Ni C., Xie Q., Hu M., Wang F., Hu J. (2017). TMSCF_3_ as a Convenient Source of CF_2_=CF_2_ for Pentafluoroethylation, (Aryloxy)tetrafluoroethylation, and Tetrafluoroethylation. Angew. Chem. Int. Ed..

[49] Future Market Insights (2024). https://www.futuremarketinsights.com/reports/fluoropolymers-market.

[50] Puts G.J., Crouse P., Ameduri B.M. (2019). Polytetrafluoroethylene: Synthesis and Characterization of the Original Extreme Polymer. Chem. Rev..

[51] Ameduri B., Boutevin B. (2000). Copolymerization of Fluorinated Monomers: Recent Developments and Future Trends. J. Fluor. Chem..

[52] Smith D.W., Iacono S.T., Iyer S.S. (2014).

[53] Ohashi M., Ogoshi S. (2016). Transition-Metal Mediated Transformations of Tetrafluoroethylene into Various Polyfluorinated Organic Compounds. J. Syn. Org. Chem. Jpn..

[54] Ogoshi S., Doi R., Zhou Y. (2023). Transformation of Tetrafluoroethylene Using Transition-Metal Complexes. Synthesis.

[55] Sprague L., Graham D., Ferstandig L. (2001). Production of Aliphatic Fluorocarbons. WO Patent WO2001007384.

[56] Iikubo Y., Hedrick V., Brandstadter S.M., Cohn M. (2004). Materials and Methods for the Conversion of Hydrofluorocarbons. US Patent US20040127757.

[57] Fujihira Y., Hirano K., Ono M., Mimura H., Kagawa T., Sedgwick D.M., Fustero S., Shibata N. (2021). Pentafluoroethylation of Carbonyl Compounds by HFC-125 via the Encapsulation of the K Cation with Glymes. J. Org. Chem..

[58] Ono M., Sumii Y., Fujihira Y., Kagawa T., Mimura H., Shibata N. (2021). Pentafluoroethylation of Carbonyl Compounds Using HFC-125 in a Flow Microreactor System. J. Org. Chem..

[59] Fujihira Y., Iwasaki H., Sumii Y., Adachi H., Kagawa T., Shibata N. (2022). Continuous-Flow Synthesis of Perfluoroalkyl Ketones via Perfluoroalkylation of Esters Using HFC-23 and HFC-125 under a KHMDS-Triglyme System. Bull. Chem. Soc. Jpn..

[60] Sumii Y., Iwasaki H., Fujihira Y., Mahmoud E.M., Adachi H., Kagawa T., Cahard D., Shibata N. (2022). KHMDS/Triglyme Cryptate as an Alternative to Phosphazene Base in Stereodivergent Pentafluoroethylation of *N*-Sulfinylimines Using HFC-125. J. Org. Chem..

[61] Dixon D.A., Fukunaga T., Smart B.E. (1986). Structures and Stabilities of Fluorinated Carbanions. Evidence for Anionic Hyperconjugation. J. Am. Chem. Soc..

[62] Ameduri B. (2011). Controlled Radical (Co)polymerization of Fluoromonomers. Macromolecules.

[63] Ameduri B. (2018). Fluoropolymers: The Right Material for the Right Applications. Chem. Eur J..

[64] Zhang Z., Chen K., Ameduri B., Chen M. (2023). Fluoropolymer Nanoparticles Synthesized via Reversible-Deactivation Radical Polymerizations and Their Applications. Chem. Rev..

[65] Ameduri B., Hori H. (2023). Recycling and the end of life assessment of fluoropolymers: recent developments, challenges and future trends. Chem. Soc. Rev..

[66] Liang L., Wen T., Xin J., Su C., Song K., Zhao W., Liu H., Su G. (2023). Fluoropolymer: A Review on Its Emulsion Preparation and Wettability to Solid-Liquid Interface. Molecules.

[67] Jaye J.A., Sletten E.M. (2019). Modular and Processable Fluoropolymers Prepared via a Safe, Mild, Iodo–Ene Polymerization. ACS Cent. Sci..

[68] Boswell B.R., Mansson C.M.F., Cox J.M., Jin Z., Romaniuk J.A.H., Lindquist K.P., Cegelski L., Xia Y., Lopez S.A., Burns N.Z. (2021). Mechanochemical synthesis of an elusive fluorinated polyacetylene. Nat. Chem..

[69] Zhao Y., Chen Y., Zhou H., Zhou Y., Chen K., Gu Y., Chen M. (2023). Controlled radical copolymerization of fluoroalkenes by using light-driven redox-relay catalysis. Nat. Synth..

[70] Tashiro K., Akiyama M., Kashiwagi K., Okazoe T. (2023). The Fluorocarbene Exploit: Enforcing Alternation in Ring-Opening Metathesis Polymerization. J. Am. Chem. Soc..

[71] Watanabe K., Tomar D., Ikuno K., Tsunekawa H., Inoue K.i., Ye S. (2025). Elucidation of the Adhesion Mechanism for PVDF-Based Binders on the Current Collector of the Cathode in Lithium-Ion Batteries. ACS Appl. Polym. Mater..

[72] He F., Gao Y., Jin K., Wang J., Sun J., Fang Q. (2016). Conversion of a Biorenewable Plant Oil (Anethole) to a New Fluoropolymer with Both Low Dielectric Constant and Low Water Uptake. ACS Sustain. Chem. Eng..

[73] Dong Y., Wang Z., Li C. (2017). Controlled radical fluorination of poly(meth)acrylic acids in aqueous solution. Nat. Commun..

[74] Xu G., Pan J., Zou X., Jin Z., Zhang J., Fang P., Zhang Q., Sun Z., Yan F. (2023). High-performance Poly(biphenyl piperidinium) Type Anion Exchange Membranes with Interconnected Ion Transfer Channels: Cooperativity of Dual Cations and Fluorinated Side Chains. Adv. Funct. Mater..

[75] Zhou Q., Li K., Wang K., Hong W., Chen J., Chai J., Yu L., Si Z., Li P. (2024). Fluoroamphiphilic polymers exterminate multidrug-resistant Gram-negative ESKAPE pathogens while attenuating drug resistance. Sci. Adv..

[76] Yang Z., Zhu Y., Tan X., Gunjal S.J.J., Dewapriya P., Wang Y., Xin R., Fu C., Liu K., Macintosh K. (2024). Fluoropolymer sorbent for efficient and selective capturing of per- and polyfluorinated compounds. Nat. Commun..

[77] Sunagawa D.E., Ishida N., Iwamoto H., Ohashi M., Fruit C., Ogoshi S. (2021). Synthesis of Fluoroalkyl Sulfides via Additive-Free Hydrothiolation and Sequential Functionalization Reactions. J. Org. Chem..

[78] Ohashi M., Kamura R., Doi R., Ogoshi S. (2013). Preparation of Trifluorovinyl Compounds by Lithium Salt Promoted Monoalkylation of Tetrafluoroethylene. Chem. Lett..

[79] Kosobokov M., Cui B., Balia A., Matsuzaki K., Tokunaga E., Saito N., Shibata N. (2016). Importance of a Fluorine Substituent for the Preparation of *meta*- and *para*-Pentafluoro-λ^6^-sulfanyl-Substituted Pyridines. Angew. Chem. Int. Ed..

[80] Das P., Niina K., Hiromura T., Tokunaga E., Saito N., Shibata N. (2018). An Eccentric Rod-like Linear Connection of Two Heterocycles: Synthesis of Pyridine *trans*-tetrafluoro-λ^6^-sulfanyl Triazoles. Chem. Sci..

[81] Saidalimu I., Liang Y., Niina K., Tanagawa K., Saito N., Shibata N. (2019). Synthesis of Aryl and Heteroaryl Tetrafluoro-λ^6^-sulfanyl Chlorides from Diaryl Disulfides Using Trichloroisocyanuric Acid and Potassium Fluoride. Org. Chem. Front..

[82] Yasuo E., Aikawa K., Nozaki K., Okazoe T. (2023). Fluoroalkylated Hypervalent Sulfur Fluorides: Radical Addition of Arylchlorotetrafluoro-λ^6^-sulfanes to Tetrafluoroethylene. Chem. Sci..

[83] Petrov V.A., Swearingen S., Hong W., Chris Petersen W. (2001). 1,1,2,2-Tetrafluoroethyl-N,N-dimethylamine: a New Selective Fluorinating Agent. J. Fluor. Chem..

[84] Parmar K., Gaitonde S., Sogani S. (2015). Single Pot Process to Prepare Ethyl 2,2-difluoroacetic Acid. Patent IN2014DE01501.

[85] Koh M., Sakata H., Nakazawa A., Kagawa M., Nakazono A. (2010). Electrolytic Solutions and Electrochemical Devices and Lithium-ion Secondary Batteries Therewith. JP Patent JP2010146740.

[86] Wada S., Aida S., Yamamoto H. (2011). Manufacture of Fluoropolymers Using Hydrofluoroalkyl Ether Chain Transfer Agents. JP Patent JP2011032363.

[87] Abe T., Nakamura K., Fujii H. (2016). Laminated tubes for automobile fuels. JP Patent JP2016034711.

[88] Sumii Y., Shibata N. (2023). Current State of Microflow Trifluoromethylation Reactions. Chem. Rec..

[89] Fu W.C., MacQueen P.M., Jamison T.F. (2021). Continuous flow strategies for using fluorinated greenhouse gases in fluoroalkylations. Chem. Soc. Rev..

[90] Han S., Kashfipour M.A., Ramezani M., Abolhasani M. (2020). Accelerating gas–liquid chemical reactions in flow. Chem. Commun..

[91] Hirano K., Gondo S., Punna N., Tokunaga E., Shibata N. (2019). Gas/Liquid-Phase Micro-Flow Trifluoromethylation using Fluoroform: Trifluoromethylation of Aldehydes, Ketones, Chalcones, and *N*-Sulfinylimines. ChemistryOpen.

[92] Inoue M., Sumii Y., Shibata N. (2020). Contribution of Organofluorine Compounds to Pharmaceuticals. ACS Omega.

[93] Ogawa Y., Tokunaga E., Kobayashi O., Hirai K., Shibata N. (2020). Current Contributions of Organofluorine Compounds to the Agrochemical Industry. iScience.

[94] Prakash G.K.S., Jog P.V., Batamack P.T.D., Olah G.A. (2012). Taming of Fluoroform: Direct Nucleophilic Trifluoromethylation of Si, B, S, and C Centers. Science.

[95] Frisch M.J., Trucks G.W., Schlegel H.B., Scuseria G.E., Robb M.A., Cheeseman J.R., Scalmani G., Barone V., Petersson G.A., Nakatsuji H. (2016).

[96] Legault C.Y. (2020). http://www.cylview.org.

[97] Raghavanpillai A., Burton D.J. (2004). Room Temperature Preparation of Trifluoroethenylzinc Reagent by Metalation of the Readily Available Halocarbon HFC-134a and an Efficient, Economically Viable Synthesis of 1,2,2-Trifluorostyrenes. J. Org. Chem..

[98] England D.C., Melby L.R., Dietrich M.A., Lindsey R.V. (1960). Nucleophilic Reactions of Fluoroölefins. J. Am. Chem. Soc..

[99] Djukanovic D., Heinz B., Mandrelli F., Mostarda S., Filipponi P., Martin B., Knochel P. (2021). Continuous Flow Acylation of (Hetero)aryllithiums with Polyfunctional N,N-Dimethylamides and Tetramethylurea in Toluene. Chem. Eur J..

[100] Thoai N. (1988). Transformation D'oxydes D'alkyles et de Polyfluoroalkyles par les Acides de Lewis. Influence des Radicaux Alkyls. J. Fluorine Chem..

[101] Chai J.-D., Head-Gordon M. (2008). Long-Range Corrected Hybrid Density Functionals with Damped Atom–Atom Dispersion Corrections. Phys. Chem. Chem. Phys..

[102] Weigend F., Ahlrichs R. (2005). Balanced Basis Sets of Split Valence, Triple Zeta Valence and Quadruple Zeta Valence Quality for H to Rn: Design and Assessment of Accuracy. Phys. Chem. Chem. Phys..

[103] Marenich A.V., Cramer C.J., Truhlar D.G. (2009). Universal Solvation Model Based on Solute Electron Density and on a Continuum Model of the Solvent Defined by the Bulk Dielectric Constant and Atomic Surface Tensions. J. Phys. Chem. B.

[104] Fukui K. (1981). The Path of Chemical Reactions - the IRC Approach. Acc. Chem. Res..

