# Supplementary material for: Repurposing HFC-125 to tetrafluoroethylene: A step toward a more sustainable fluoropolymer feedstock strategy
Source: iScience. 2025 May 3;28(6):112580. doi: 10.1016/j.isci.2025.112580 (PMC12148618; doi:10.1016/j.isci.2025.112580)
Supplement: Document S1. Figures S1–S11, Tables S1 and S2, and Data S1 and S2 [file mmc1.pdf]

iScience, Volume *28*

Supplemental information

Repurposing HFC-125 to tetrafluoroethylene:

A step toward a more sustainable

fluoropolymer feedstock strategy

Hiroto Iwasaki, Naoyuki Hoshiya, Yosuke Kishikawa, Jorge Escorihuela, and Norio Shibata

Supplementary Information

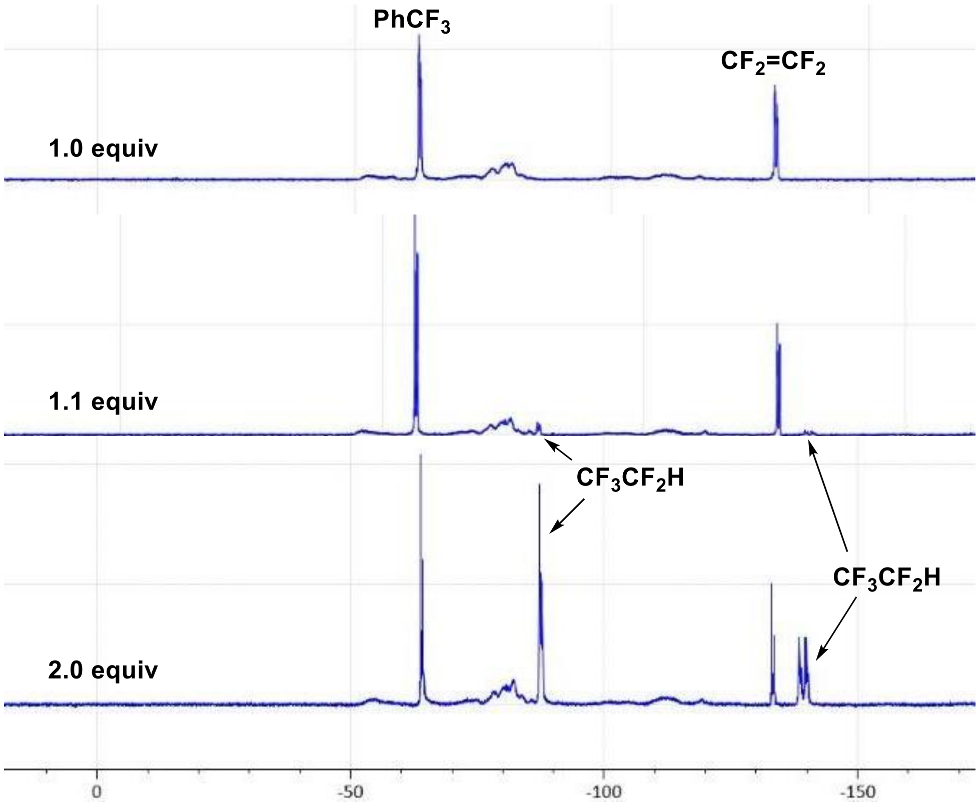

Figure S1. Examination of the equivalent of HFC-125.[a] Related to Figure 2.
[a]Reaction conditions: HFC−125, KHMDS (0.6 mmol, 1.0 equiv), and $PhCF_3$ as the internal standard in Toluene (2.0 mL). Reaction mixture was stirred at room temperature for 1 h.

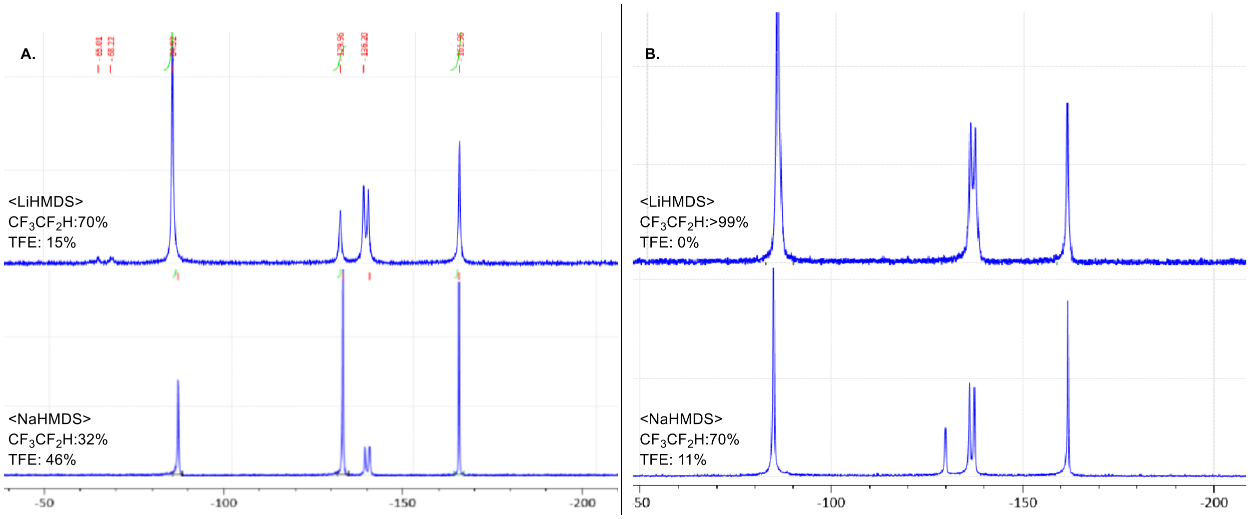

Figure S2. Comparison of long-time reactions using LiHMDS or NaHMDS.[a] Related to Figure 2.
(A) Reaction at room temperature. (B) Reaction at −50 °C.
[a]Reaction conditions: HFC−125 (1.0 equiv), LiHMDS or NaHMDS (0.6 mmol, 1.0 equiv), and C_6F_6 (0.1 mmol) as the internal standard in Toluene (2.0 mL). Reaction mixture was stirred at room temperature (A) or −50 °C (B) for 12 h.

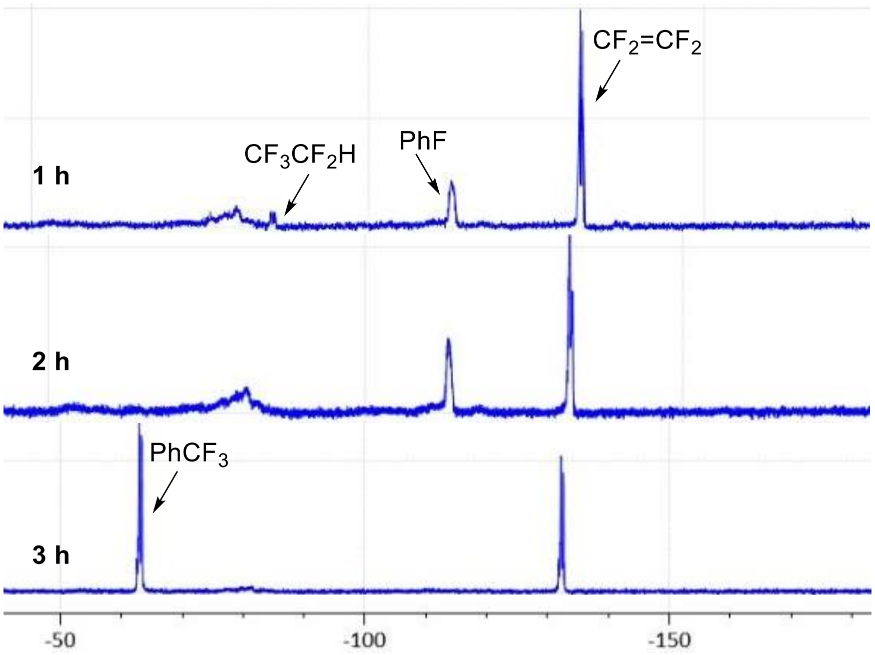

Figure S3. Optimization of reaction time.[a] Related to Figure 2.

[a]Reaction conditions: HFC−125 (1.0 equiv), KHMDS (0.6 mmol, 1.0 equiv), and PhF (1 h and 2 h) or PhCF$_3$ (3 h) as the internal standard in Toluene (2.0 mL). Reaction mixture was stirred at −50 °C for each time.

Table S1. Verification of TFE generation under various conditions.[a] Related to STAR Methods.

Entry	Base (equiv)	Additive (equiv)	Solvent	Temp. (°C)	Time (h)	Generation of TFE
1	KHMDS (1.0)	-	THF	-50	3	○
2	KHMDS (1.0)	-	Toluene/Triglyme (1/1)	-50	1	×
3	LDA (2.0)	-	Toluene	-50	1	○
4	DBU (2.0)	-	Toluene	-50	1	×
5	NaH (2.0)	-	Toluene	-50	3	×
6	NaH (5.0)	-	Toluene/*t*BuOH (3/1)	-50	1	×
7	*t*BuOK (2.0)	-	Triglyme	-40	1	×
8	KHMDS (1.0)	-	*n*Hexane	25	1	○
9	KHMDS (1.0)	-	Toluene/EtOAc	25	1	×
10	KHMDS (1.0)	-	EtOAc	25	1	×
11	*t*BuOK (1.0)	-	THF	25	1	×
12	*t*BuOK (2.0)	-	DMSO	25	1	×
13	*t*BuOK (2.0)	-	Triglyme	25	1	×
14	*t*BuOK (2.0)	18-crown-6 (0.5)	Toluene	25	1	×
15	*t*BuOK (1.0)	TBAI (1.0)	Toluene	25	1	○
16	*t*BuOK (2.0)	TMAI (1.0)	Toluene	25	1	×
17	*t*BuOK (2.0)	TEAI (1.0)	Toluene	25	1	×
18	KOH (1.0)	-	DMF	25	1	×
19	KOH (1.0)	-	DMSO	25	1.5	×
20	KOH (2.0)	TBAI (1.0)	Toluene	25	1	×
21	KOH (2.0)	TBAI (1.0)	*n*Hexane	25	1	×
22	KOH (5.0)	TBAI (1.0)	Toluene/*t*BuOH (3/1)	25	1	×

23	KOH (5.0)	*t*BuOH (5.0), TBAI (1.0)	Toluene	25	1	×
24	KOH (5.0)	TBAI (1.0)	Toluene/H_2O (3/1)	25	1	×
25	NaOH (1.0)	-	DMSO	25	1	×
26	NaOH (2.0)	TBAI (1.0)	Toluene	25	1	×
27	NaOH (2.0)	TBAI (1.0)	*n*Hexane	25	1	×
28	NaH (5.0)	-	Toluene/*t*BuOH (3/1)	25	1	×
29	NaH (2.0)	TBAI (1.0)	Toluene	25	1	×

[a]Reaction conditions: HFC−125 (0.6 mmol), Base, Additives and PhF or $PhCF_3$ or C_6F_6 as the internal standard in Solvent (2.0 mL).

Table S2. Integral value of pure TFE for calibration curve preparation.[a] Related to Table 1.

TFE (mmol)	Integral Value	Average	Standard Deviation (SD)
0.00	0	0	0.00
0.01	41.38	41.89	1.81
0.01	40.68		
0.01	41.38		
0.01	45.08		
0.01	40.93		
0.02	85.78	87.06	1.80
0.02	88.33		
0.03	140.40	138.32	2.94
0.03	136.24		

[a]**Procedure:** The NMR tube was filled with 0.7 mL of toluene−*d*8 and 1.14 μL (0.01 mmol) of hexafluorobenzane as an internal standard, covered with a septum, and sealed with parafilm. The solution was frozen in liquid nitrogen and vacuumed. Specimens of TFE were transferred from cylinders to a balloon and injected using a gastight syringe. While the lower part of the tube was immersed in liquid nitrogen, the upper part of the tube was warmed by hand to melt HFC−125 down to the solution interface. The mixture was transferred to a low temperature methanol bath at −90 °C and left for 1 hour. ^{19}F−NMR was measured while keeping the temperature as low as possible.

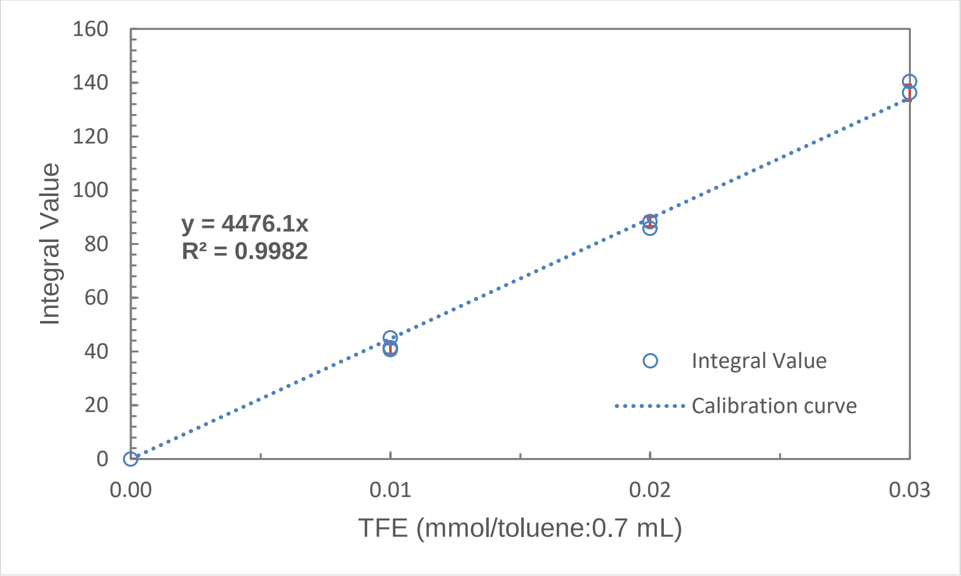

Figure S4. TFE calibration curve. Related to Table 1. Each data point represents the measurement ± SD from multiple replicate measurements (n = 5 for 0.01 mmol, n = 2 for 0.02 mmol, and n = 2 for 0.03 mmol). The Calibration curve is shown with error bars indicating measurement variability.

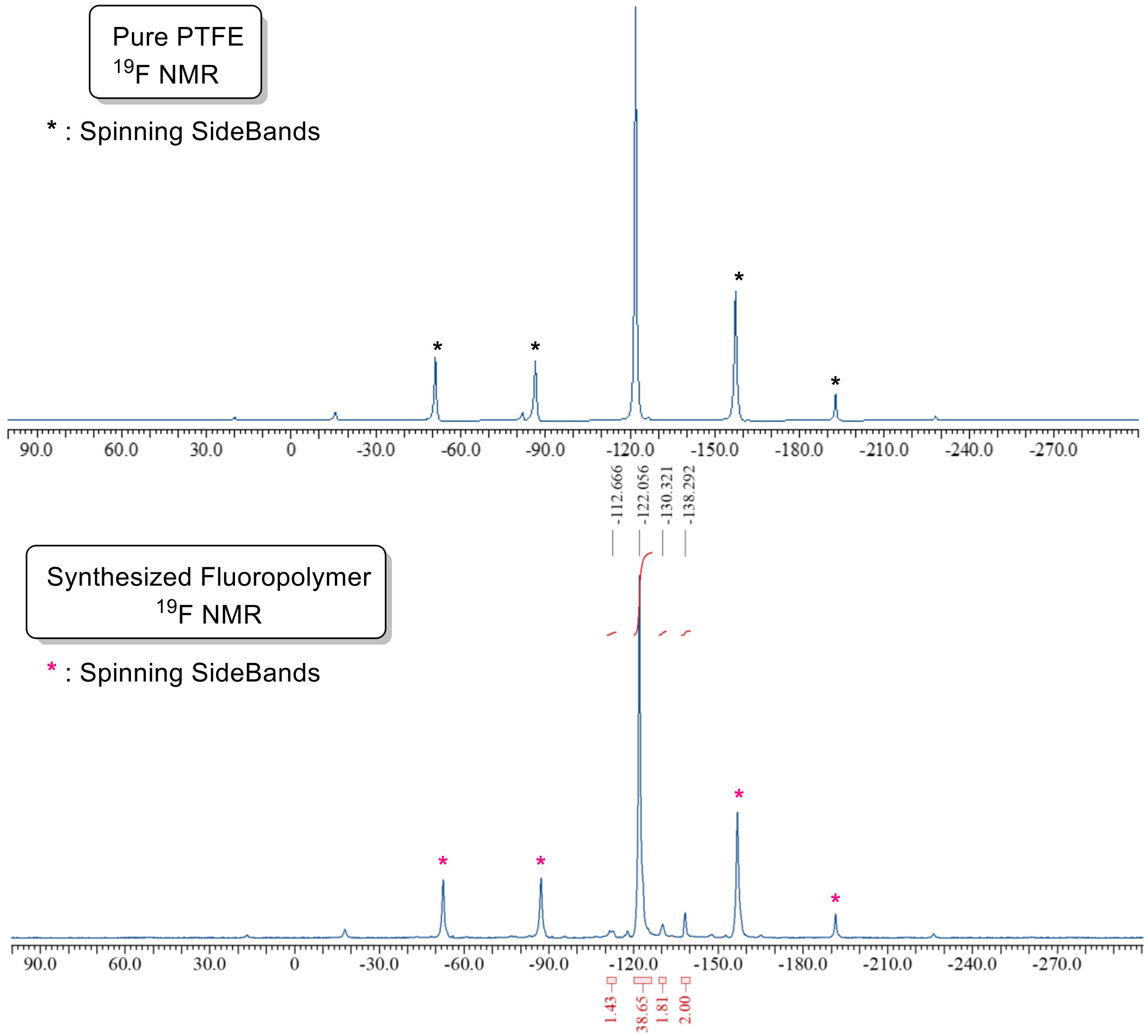

Figure S5. ^{19}F NMR chart for comparison with the NMR spectrum of pure PTFE. Related to Figure 4F.

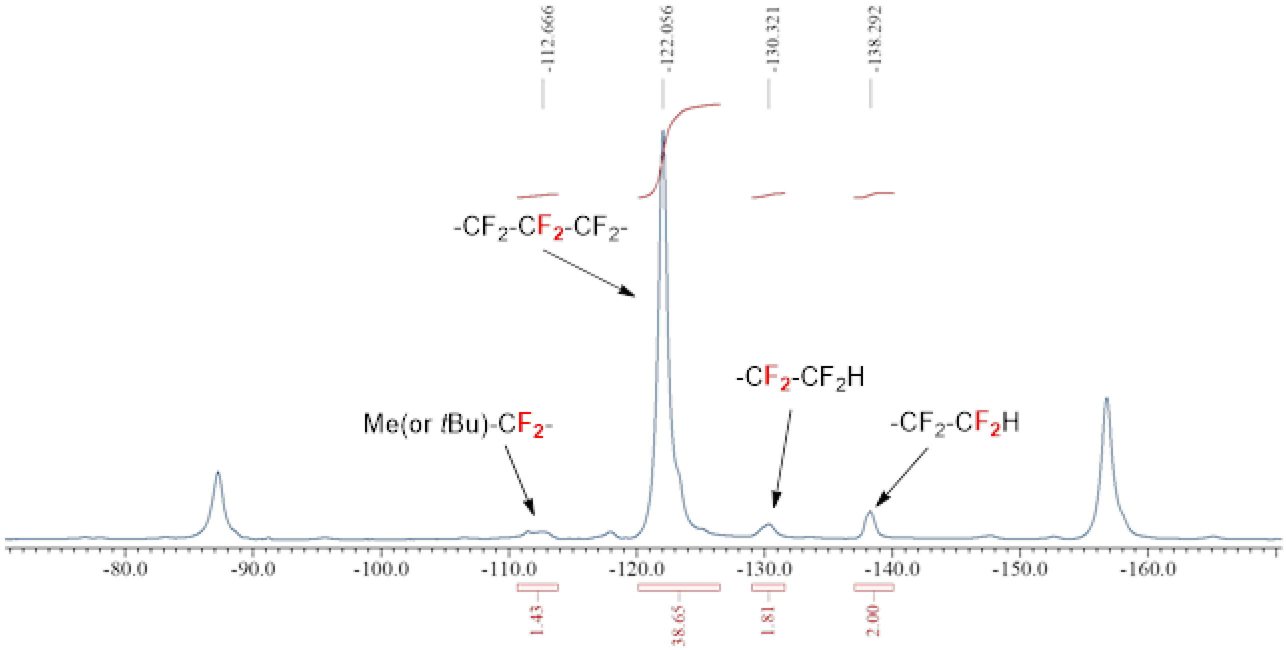

Figure S6. ^{19}F NMR chart for determination of degree of polymerization. Related to Figure 4F.

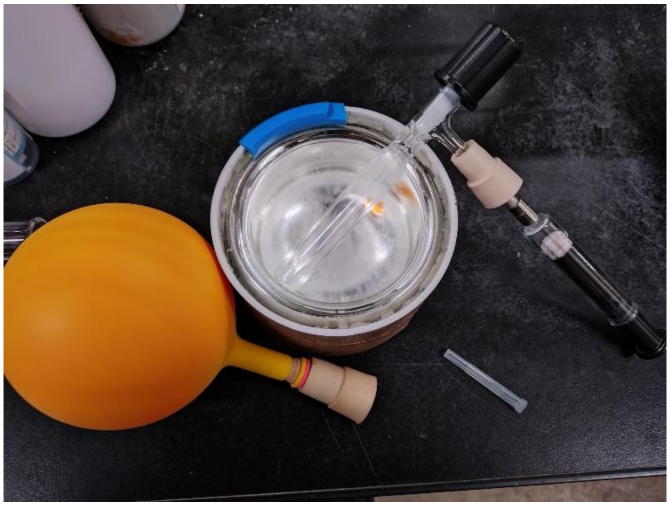

Figure S7. Addition of HFC-125 by a gas tight syringe. Related to STAR Methods.

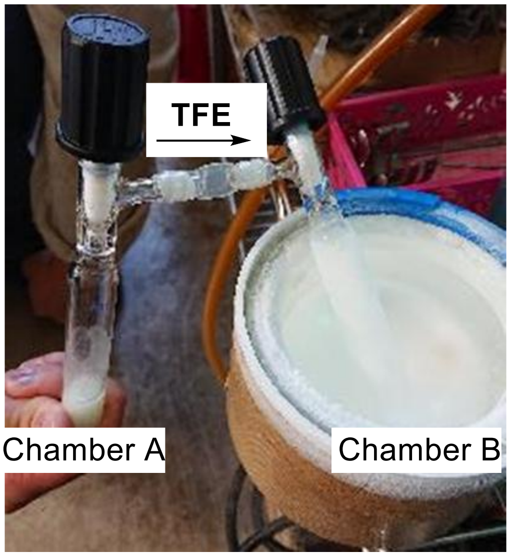

Figure S8. Transfer of the generated TFE. Related to STAR Methods.

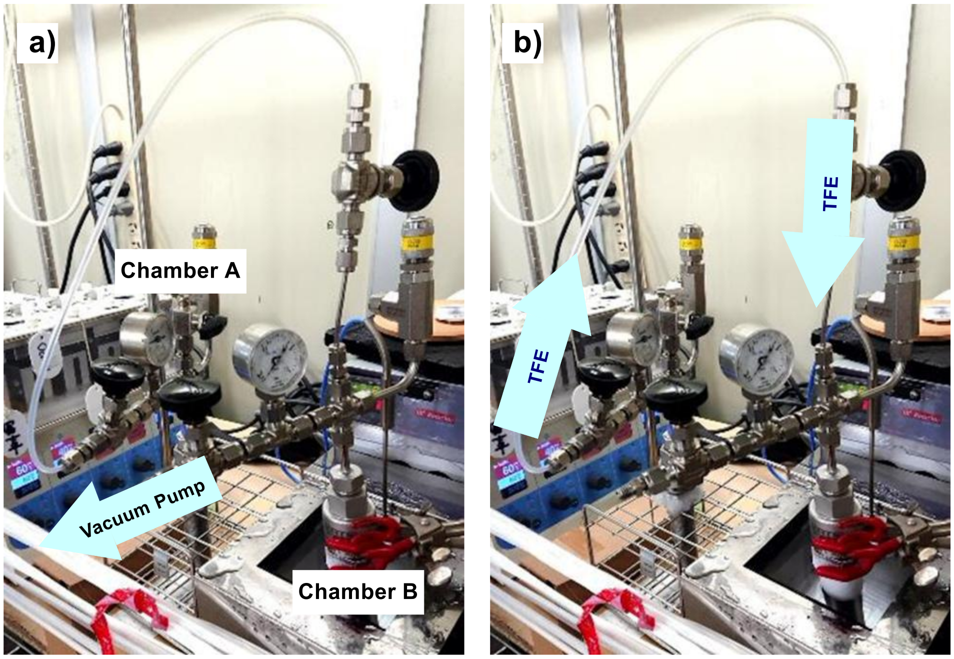

Figure S9. Transfer of the produced TFE using an autoclave. Related to STAR Methods.

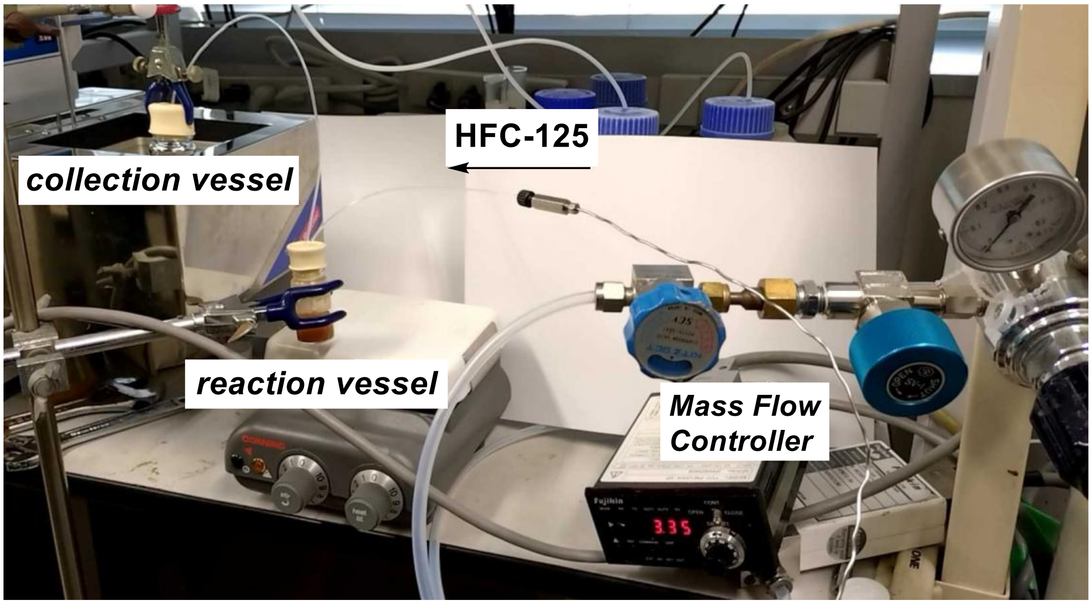

Figure S10. TFE synthesis flow line system. Related to STAR Methods.

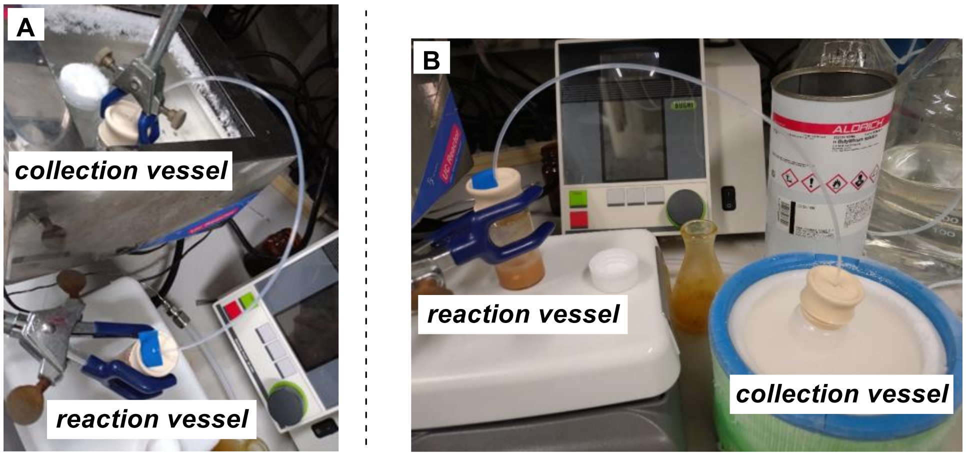

Figure S11. Expulsion of gases. Related to STAR Methods.

Data S1. Computational data including energies and cartesian coordinates of stationary points. Related to Figure 3**.**

All density functional theory (DFT) calculations were carried out using the Gaussian16 package.[1] All the structures were optimized using the long-range corrected hybrid ωB97xD density functional[2] in combination with the Def2TZVP basis set.[3] The effect of the solvent was mimicked by applying the SMD model using ether as solvent.[4] The nature of the stationary points was confirmed by frequency calculations analysis at the same level of theory (minima were characterized by no imaginary frequencies, whereas transition states had one imaginary frequency). Transition states were further verified by relaxing the imaginary frequency towards the reactant and the product and by means of IRC calculations.[5] 3D structures of optimized stationary points were represented using the CYLview 1.0 program.[6]

HFC-125

E(electronic) = -576.127342254
Zero-point correction= 0.037856 (Hartree/Particle)
Thermal correction to Energy= 0.043937
Thermal correction to Enthalpy= 0.044882
Thermal correction to Gibbs Free Energy= 0.007100
Sum of electronic and zero-point Energies= -576.089486
Sum of electronic and thermal Energies= -576.083405
Sum of electronic and thermal Enthalpies= -576.082461
Sum of electronic and thermal Free Energies= -576.120243

6	0.616829000	-0.000083000	-0.005318000
9	1.227943000	-1.080349000	-0.486903000
9	1.227971000	1.080130000	-0.487397000
9	0.733831000	0.000241000	1.317395000
6	-0.860363000	-0.000024000	-0.440699000
9	-1.461304000	-1.092731000	0.061977000
9	-1.461168000	1.092772000	0.062080000
1	-0.944256000	0.000072000	-1.528268000

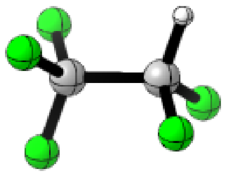

KHMDS

E(electronic) = -1473.37467905
Zero-point correction= 0.226949 (Hartree/Particle)
Thermal correction to Energy= 0.245150
Thermal correction to Enthalpy= 0.246094
Thermal correction to Gibbs Free Energy= 0.180675
Sum of electronic and zero-point Energies= -1473.147730
Sum of electronic and thermal Energies= -1473.129529
Sum of electronic and thermal Enthalpies= -1473.128585
Sum of electronic and thermal Free Energies= -1473.194004

7	-0.000004000	0.194695000	-0.000179000
14	-1.526520000	-0.483249000	0.001571000
14	1.526482000	-0.483315000	-0.001618000
6	-1.872862000	-1.665447000	1.437545000
1	-1.688579000	-1.179684000	2.398834000
1	-2.905487000	-2.023906000	1.431946000
1	-1.217538000	-2.538269000	1.382027000
6	-2.840252000	0.885090000	0.138264000
1	-2.795522000	1.573160000	-0.712361000
1	-3.849495000	0.467344000	0.154172000
1	-2.719543000	1.467171000	1.057359000
6	-1.968584000	-1.443582000	-1.567810000
1	-2.993796000	-1.821951000	-1.540117000
1	-1.866339000	-0.812572000	-2.454286000

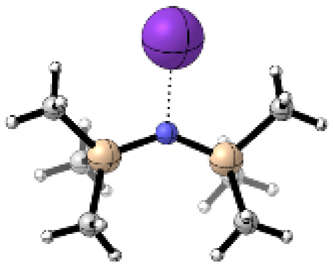

1	-1.300433000	-2.298331000	-1.697175000
6	1.873095000	-1.665356000	-1.437656000
1	1.217564000	-2.538046000	-1.382492000
1	1.689236000	-1.179404000	-2.398931000
1	2.905641000	-2.024037000	-1.431769000
6	1.968160000	-1.443862000	1.567743000
1	1.865291000	-0.813100000	2.454323000
1	1.300264000	-2.298885000	1.696608000
1	2.993521000	-1.821855000	1.540415000
6	2.840307000	0.884983000	-0.137858000
1	2.719781000	1.467228000	-1.056875000
1	2.795491000	1.572910000	0.712877000
1	3.849526000	0.467175000	-0.153685000
19	0.000094000	2.799638000	-0.000017000

INT1
E(electronic) = -1175.46879887
Zero-point correction= 0.023475 (Hartree/Particle)
Thermal correction to Energy= 0.031827
Thermal correction to Enthalpy= 0.032772
Thermal correction to Gibbs Free Energy= -0.012245
Sum of electronic and zero-point Energies= -1175.445324
Sum of electronic and thermal Energies= -1175.436971
Sum of electronic and thermal Enthalpies= -1175.436027
Sum of electronic and thermal Free Energies= -1175.481044

6	-1.176968000	-0.353115000	-0.045544000
9	-1.847065000	-0.541411000	-1.193334000
9	-0.728229000	-1.564152000	0.355323000
9	-2.091914000	0.026888000	0.870963000
6	-0.000125000	0.584677000	-0.266833000
9	-0.582463000	1.823969000	-0.517692000
9	0.515858000	0.743372000	1.082952000
19	2.614046000	-0.304598000	-0.184718000

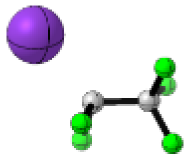

TS-I
E(electronic) = -1175.44808592
Zero-point correction= 0.022221 (Hartree/Particle)
Thermal correction to Energy= 0.030583
Thermal correction to Enthalpy= 0.031527
Thermal correction to Gibbs Free Energy= -0.012547
Sum of electronic and zero-point Energies= -1175.425865
Sum of electronic and thermal Energies= -1175.417503
Sum of electronic and thermal Enthalpies= -1175.416559
Sum of electronic and thermal Free Energies= -1175.460633
Frequency -365.5291

6	1.242435000	-0.137173000	-0.000038000
9	1.858836000	-0.524519000	-1.084594000
9	1.858862000	-0.524537000	1.084499000
9	-0.019600000	-1.488483000	0.000053000
6	0.638971000	1.112452000	0.000008000
9	-0.132375000	1.377207000	-1.101571000
9	-0.132280000	1.377101000	1.101673000
19	-2.220496000	-0.410663000	-0.000019000

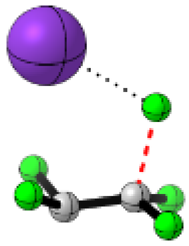

TFE
E(electronic) = -475.593526206
Zero-point correction= 0.021909 (Hartree/Particle)
Thermal correction to Energy= 0.027063
Thermal correction to Enthalpy= 0.028008
Thermal correction to Gibbs Free Energy= -0.005838
Sum of electronic and zero-point Energies= -475.571617
Sum of electronic and thermal Energies= -475.566463
Sum of electronic and thermal Enthalpies= -475.565519
Sum of electronic and thermal Free Energies= -475.599364

6	0.000000000	0.000000000	0.659069000
6	0.000000000	0.000000000	-0.659069000
9	0.000000000	1.094509000	-1.381592000
9	0.000000000	1.094509000	1.381592000
9	0.000000000	-1.094509000	-1.381592000
9	0.000000000	-1.094509000	1.381592000

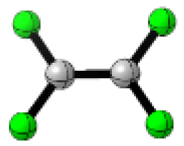

CF_3 anion
E(electronic) = -937.637845178
Zero-point correction= 0.010368 (Hartree/Particle)
Thermal correction to Energy= 0.016210
Thermal correction to Enthalpy= 0.017154
Thermal correction to Gibbs Free Energy= -0.020920
Sum of electronic and zero-point Energies= -937.627478
Sum of electronic and thermal Energies= -937.621635
Sum of electronic and thermal Enthalpies= -937.620691
Sum of electronic and thermal Free Energies= -937.658766

6	0.786089000	0.000737000	-0.296807000
9	1.642852000	1.085994000	-0.261671000
9	0.331750000	-0.021728000	1.104411000
9	1.665393000	-1.065876000	-0.289437000
19	-1.972447000	0.000529000	-0.168362000

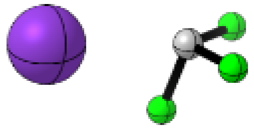

CF_2 carbene
E(electronic) = -237.737892023
Zero-point correction= 0.006988 (Hartree/Particle)
Thermal correction to Energy= 0.009976
Thermal correction to Enthalpy= 0.010921
Thermal correction to Gibbs Free Energy= -0.016389
Sum of electronic and zero-point Energies= -237.730904
Sum of electronic and thermal Energies= -237.727916
Sum of electronic and thermal Enthalpies= -237.726972
Sum of electronic and thermal Free Energies= -237.754281

6	0.000000000	0.000000000	0.591333000
9	0.000000000	1.027115000	-0.197111000
9	0.000000000	-1.027115000	-0.197111000

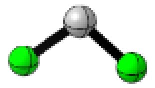

LiHMDS
E(electronic) = -880.998822167
Zero-point correction= 0.228133 (Hartree/Particle)
Thermal correction to Energy= 0.244704
Thermal correction to Enthalpy= 0.245648
Thermal correction to Gibbs Free Energy= 0.186069
Sum of electronic and zero-point Energies= -880.770689

Sum of electronic and thermal Energies= -880.754118
Sum of electronic and thermal Enthalpies= -880.753174
Sum of electronic and thermal Free Energies= -880.812753

7	0.000000000	0.759144000	-0.000009000
14	-1.518074000	0.036814000	0.004092000
14	1.518073000	0.036813000	-0.004093000
6	-1.738658000	-1.307989000	1.315846000
1	-1.523748000	-0.922488000	2.315366000
1	-2.756712000	-1.705715000	1.318300000
1	-1.056289000	-2.141984000	1.132002000
6	-2.844333000	1.342838000	0.339843000
1	-2.821069000	2.132266000	-0.417162000
1	-3.847243000	0.909604000	0.329971000
1	-2.695446000	1.812905000	1.315626000
6	-1.989575000	-0.775239000	-1.638062000
1	-2.994622000	-1.203777000	-1.603111000
1	-1.962529000	-0.049281000	-2.454723000
1	-1.291238000	-1.577454000	-1.888024000
6	1.738683000	-1.307948000	-1.315886000
1	1.056287000	-2.141933000	-1.132099000
1	1.523822000	-0.922405000	-2.315401000
1	2.756728000	-1.705697000	-1.318315000
6	1.989541000	-0.775291000	1.638046000
1	1.962444000	-0.049366000	2.454734000
1	1.291223000	-1.577538000	1.887954000
1	2.994603000	-1.203794000	1.603110000
6	2.844341000	1.342847000	-0.339776000
1	2.695468000	1.812950000	-1.315544000
1	2.821067000	2.132248000	0.417257000
1	3.847250000	0.909611000	-0.329906000
3	0.000004000	2.677254000	-0.000009000

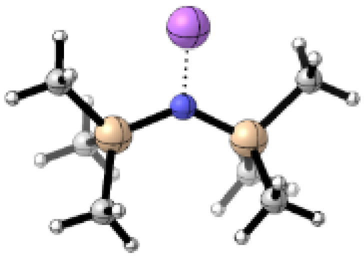

NaHMDS

E(electronic) = -1035.73341511
Zero-point correction= 0.227275 (Hartree/Particle)
Thermal correction to Energy= 0.245333
Thermal correction to Enthalpy= 0.246278
Thermal correction to Gibbs Free Energy= 0.181823
Sum of electronic and zero-point Energies= -1035.506140
Sum of electronic and thermal Energies= -1035.488082
Sum of electronic and thermal Enthalpies= -1035.487137
Sum of electronic and thermal Free Energies= -1035.551592

7	0.000000000	0.443860000	-0.000067000
14	-1.533935000	-0.225065000	0.001869000
14	1.533932000	-0.225068000	-0.001884000
6	-1.883680000	-1.396798000	1.442991000
1	-1.698570000	-0.906276000	2.401572000
1	-2.917258000	-1.752072000	1.438507000
1	-1.231095000	-2.271930000	1.392335000
6	-2.814183000	1.171689000	0.130797000
1	-2.732855000	1.861669000	-0.715588000
1	-3.837299000	0.789356000	0.133815000
1	-2.680808000	1.746530000	1.052645000
6	-1.973061000	-1.187181000	-1.564937000
1	-2.999546000	-1.561795000	-1.539577000
1	-1.865202000	-0.559579000	-2.453012000

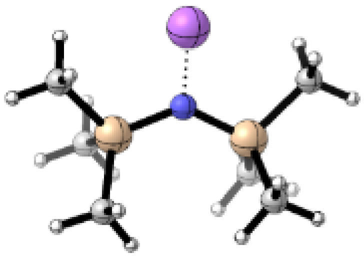

1	-1.307535000	-2.044827000	-1.688592000
6	1.883807000	-1.396687000	-1.443068000
1	1.231158000	-2.271783000	-1.392577000
1	1.698848000	-0.906064000	-2.401626000
1	2.917362000	-1.752028000	-1.438488000
6	1.972911000	-1.187310000	1.564886000
1	1.864885000	-0.559807000	2.453010000
1	1.307441000	-2.045023000	1.688366000
1	2.999430000	-1.561839000	1.539623000
6	2.814198000	1.171691000	-0.130583000
1	2.680896000	1.746623000	-1.052386000
1	2.732816000	1.861590000	0.715863000
1	3.837310000	0.789349000	-0.133567000
11	0.000010000	2.685483000	-0.000014000

INT1-Li
E(electronic) = -583.072392620
Zero-point correction= 0.024706 (Hartree/Particle)
Thermal correction to Energy= 0.032533
Thermal correction to Enthalpy= 0.033478
Thermal correction to Gibbs Free Energy= -0.008204
Sum of electronic and zero-point Energies= -583.047686
Sum of electronic and thermal Energies= -583.039859
Sum of electronic and thermal Enthalpies= -583.038915
Sum of electronic and thermal Free Energies= -583.080597

6	-0.622924000	0.001621000	0.020845000
9	-1.197371000	-1.042662000	0.648165000
9	-1.190438000	1.117710000	0.518349000
9	-0.990638000	-0.074556000	-1.269011000
6	0.875866000	0.013929000	0.292209000
9	1.350292000	-1.135678000	-0.360324000
9	1.356385000	1.068603000	-0.503319000
3	1.509426000	0.168648000	2.272311000

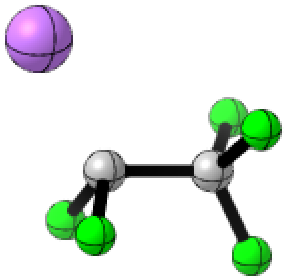

INT1-Na
E(electronic) = -737.825915204
Zero-point correction= 0.023733 (Hartree/Particle)
Thermal correction to Energy= 0.032080
Thermal correction to Enthalpy= 0.033024
Thermal correction to Gibbs Free Energy= -0.011829
Sum of electronic and zero-point Energies= -737.802182
Sum of electronic and thermal Energies= -737.793835
Sum of electronic and thermal Enthalpies= -737.792891
Sum of electronic and thermal Free Energies= -737.837744

6	-0.789501000	-0.326871000	0.000360000
9	-0.818627000	-1.131810000	-1.081624000
9	-0.821596000	-1.129571000	1.082996000
9	-1.950471000	0.354511000	-0.002058000
6	0.487008000	0.502823000	0.000902000
9	0.330106000	1.360147000	-1.105292000
9	0.327503000	1.362713000	1.103729000
11	2.564794000	-0.763602000	0.001151000

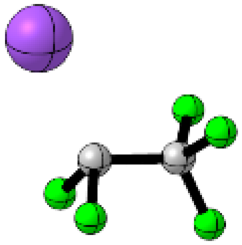

TS-I-Li
E(electronic) = -583.048977412
Zero-point correction=   0.023439 (Hartree/Particle)
Thermal correction to Energy=   0.030846
Thermal correction to Enthalpy=   0.031790
Thermal correction to Gibbs Free Energy= -0.008241
Sum of electronic and zero-point Energies=   -583.025538
Sum of electronic and thermal Energies=   -583.018132
Sum of electronic and thermal Enthalpies=   -583.017188
Sum of electronic and thermal Free Energies= -583.057218
Frequency   -387.7974

6	0.610224000	0.167099000	-0.218177000
9	1.261777000	-0.644337000	-1.006269000
9	1.369616000	1.124613000	0.221608000
9	0.603059000	-0.866947000	1.205890000
6	-0.724880000	0.488405000	-0.572804000
9	-1.458199000	-0.710045000	-0.686181000
9	-1.356638000	1.200246000	0.409726000
3	-1.029535000	-1.621601000	1.147638000

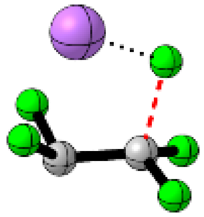

TS-I-Na
E(electronic) = -737.803372387
Zero-point correction=   0.022481 (Hartree/Particle)
Thermal correction to Energy=   0.030613
Thermal correction to Enthalpy=   0.031557
Thermal correction to Gibbs Free Energy=   -0.011344
Sum of electronic and zero-point Energies= -737.780891
Sum of electronic and thermal Energies=   -737.772759
Sum of electronic and thermal Enthalpies= -737.771815
Sum of electronic and thermal Free Energies= -737.814716
Frequency   -356.9195

6	-0.951479000	0.033430000	0.000356000
9	-1.638658000	-0.203905000	1.082959000
9	-1.640278000	-0.202161000	-1.081584000
9	-0.013228000	-1.538972000	-0.001899000
6	-0.078002000	1.121566000	0.000568000
9	0.746534000	1.162038000	1.100383000
9	0.745290000	1.163867000	-1.100033000
11	2.034540000	-0.941616000	-0.000361000

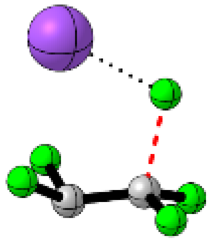

Data S2. NMR Spectra of fluorinated compounds. Related to Figure 4 and STAR Methods.

^{1}H NMR (300 MHz, Chloroform-d) of **2a**

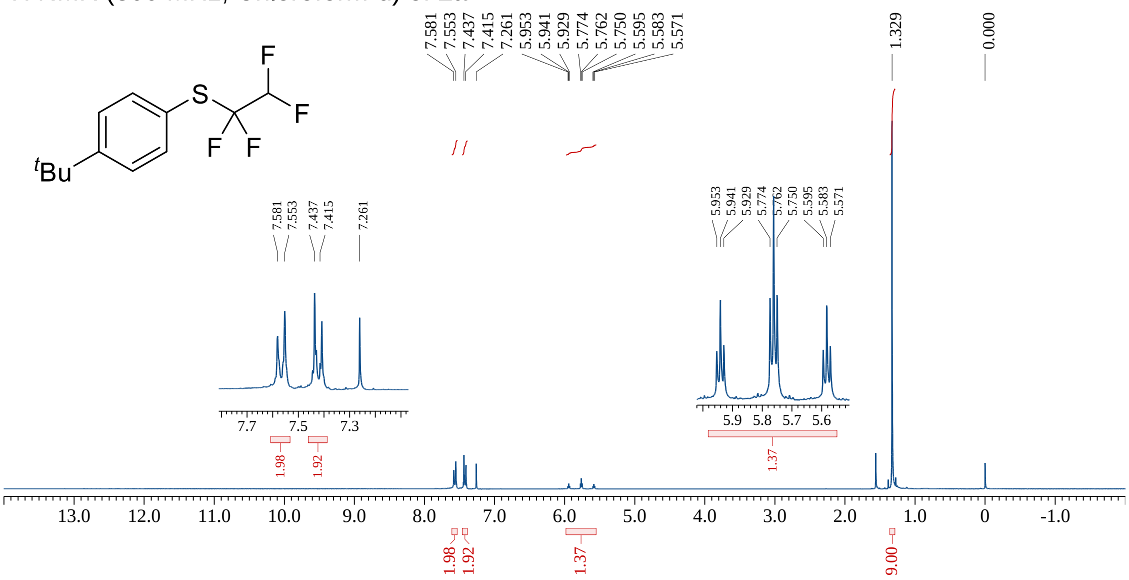

^{19}F NMR (282 MHz, Chloroform-d) of **2a**

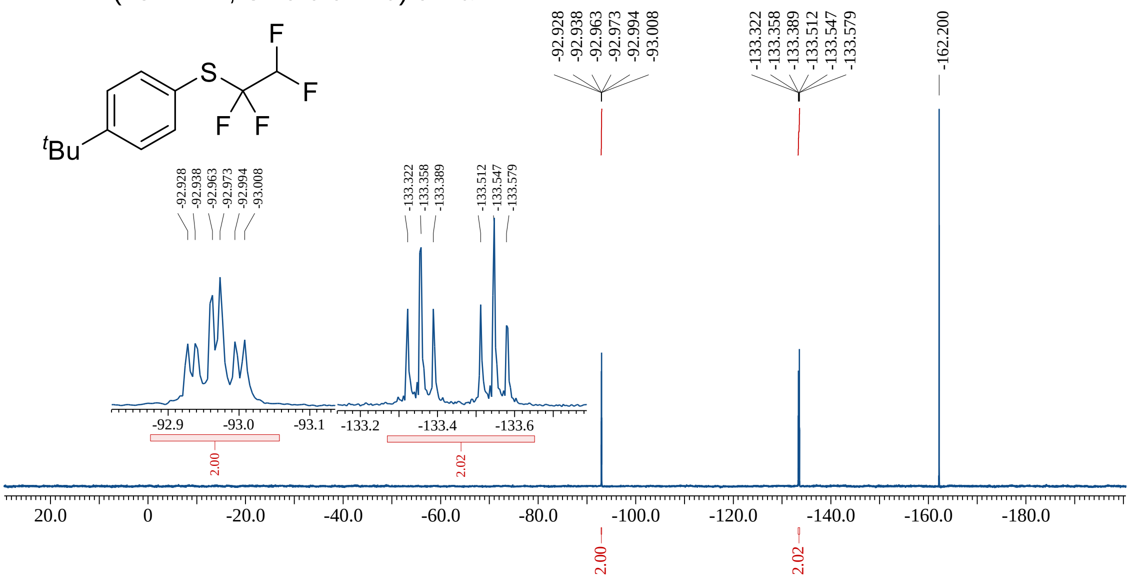

^{1}H NMR (300 MHz, Chloroform-d) of **2b**

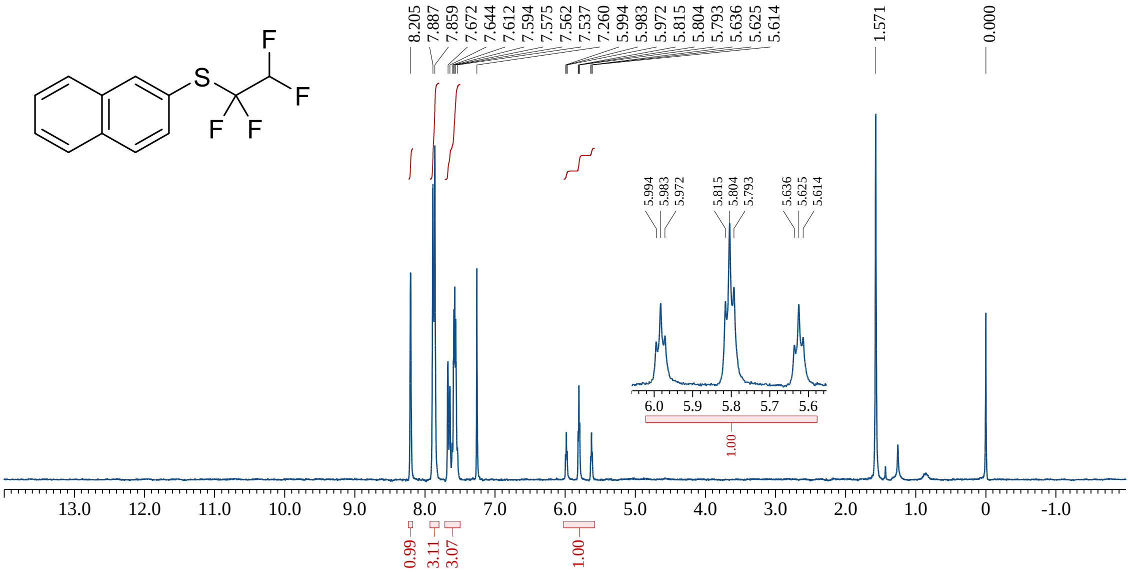

^{19}F NMR (282 MHz, Chloroform-d) of **2b**

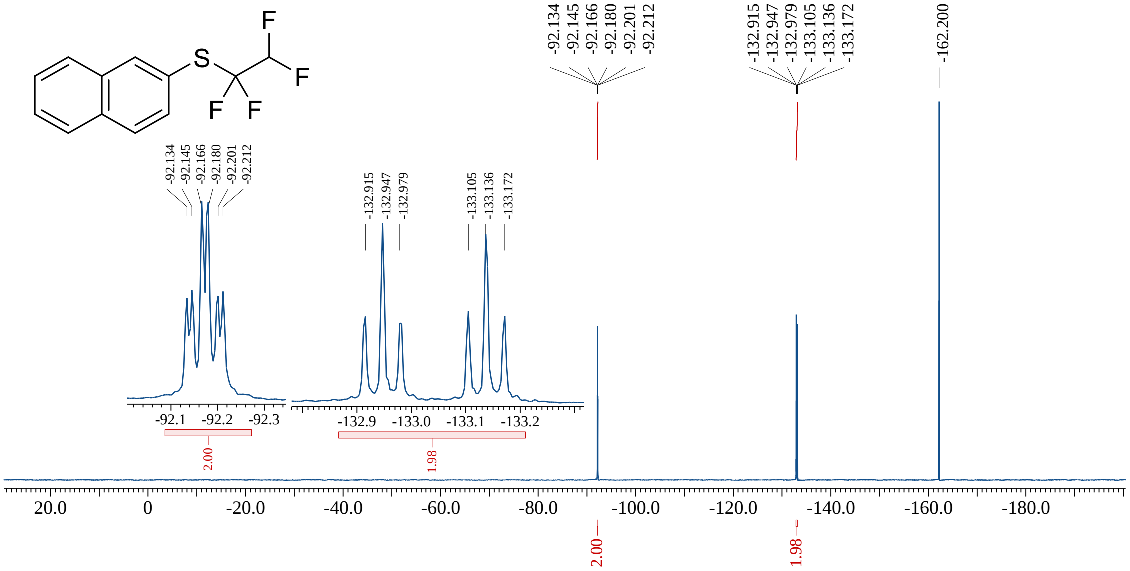

^{1}H NMR (300 MHz, Chloroform-d) of **6a**

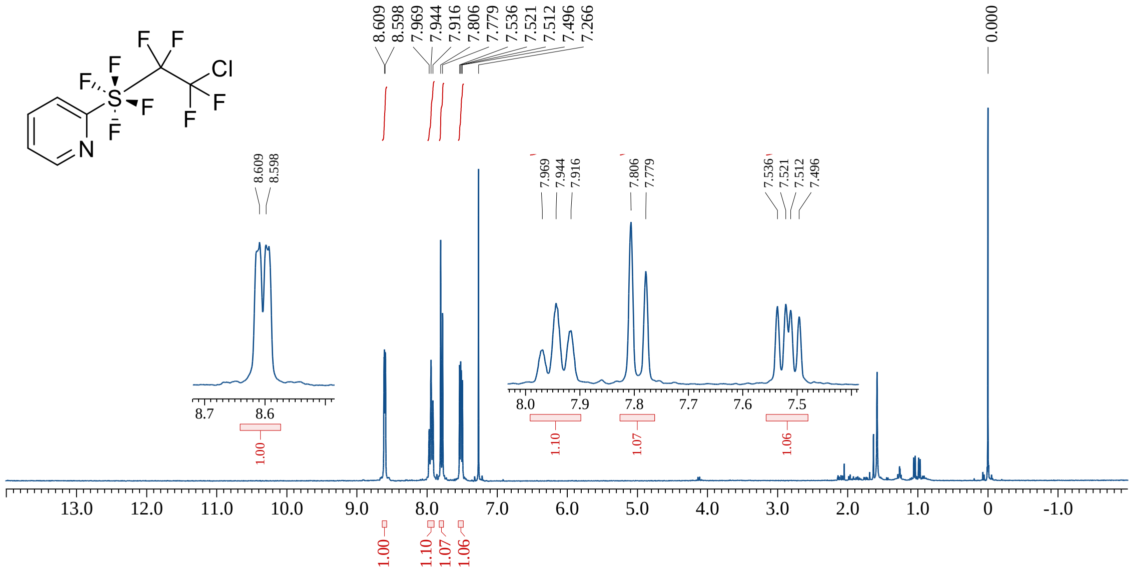

^{19}F NMR (282 MHz, Chloroform-d) of **6a**

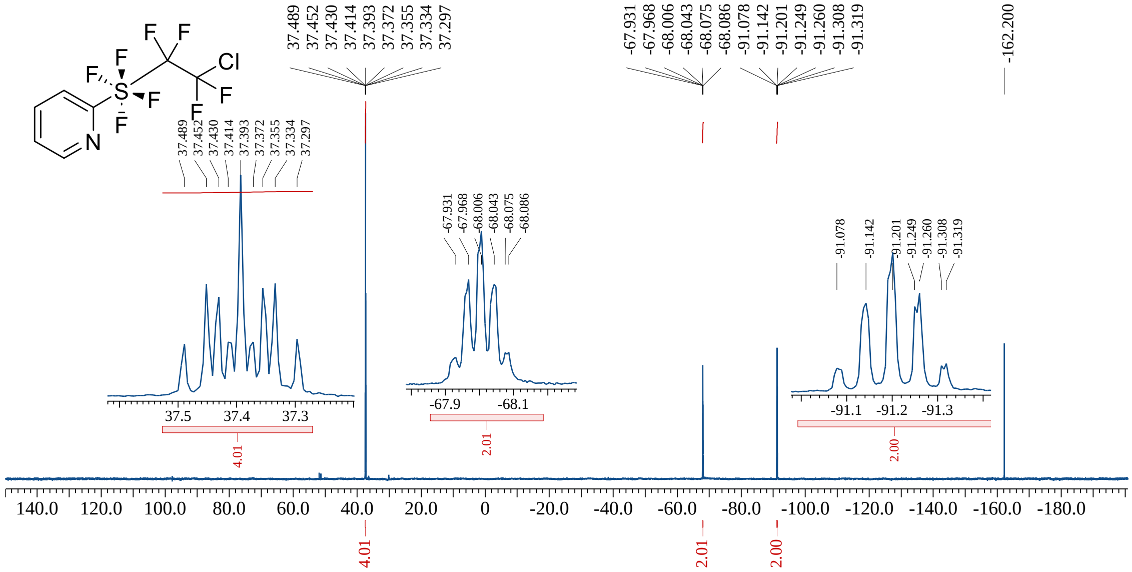

^{1}H NMR (300 MHz, Chloroform-d) of **6b**

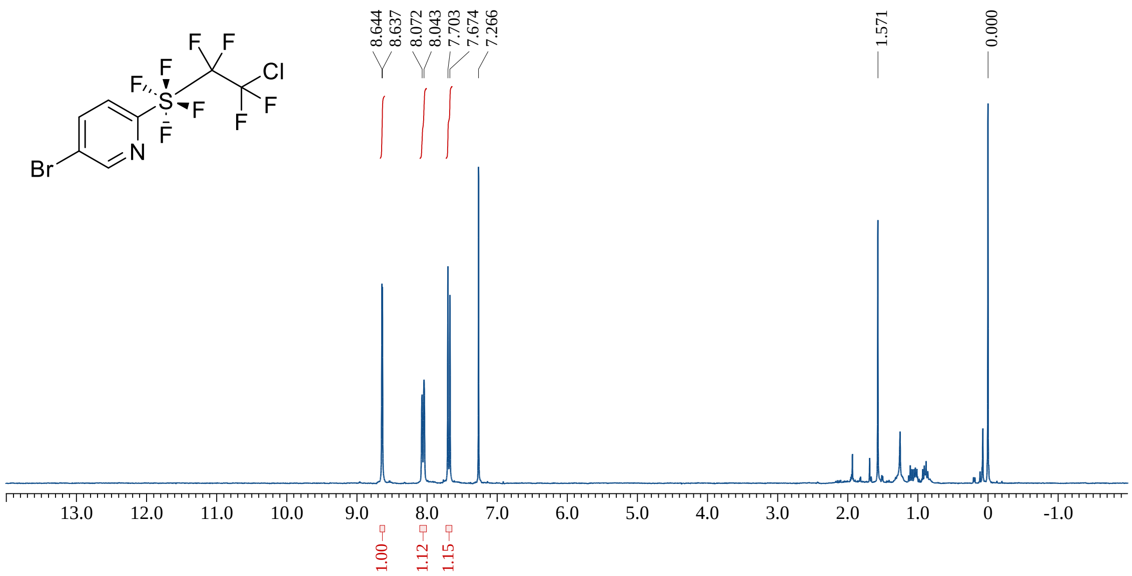

^{19}F NMR (282 MHz, Chloroform-d) of **6b**

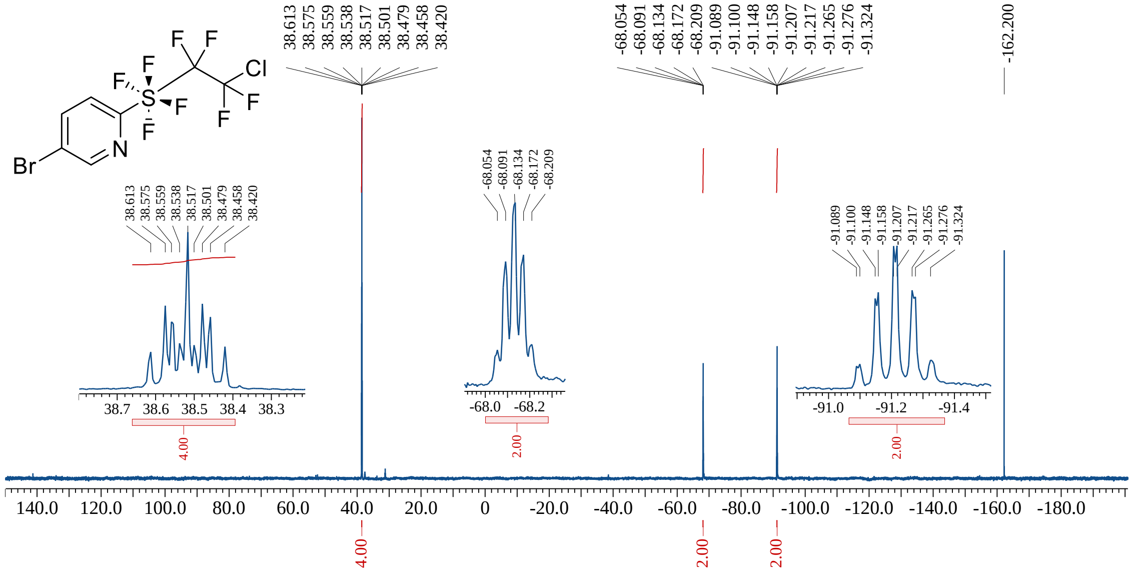

^{13}C {^{1}H} NMR (126 MHz, Chloroform-d) of **6b**

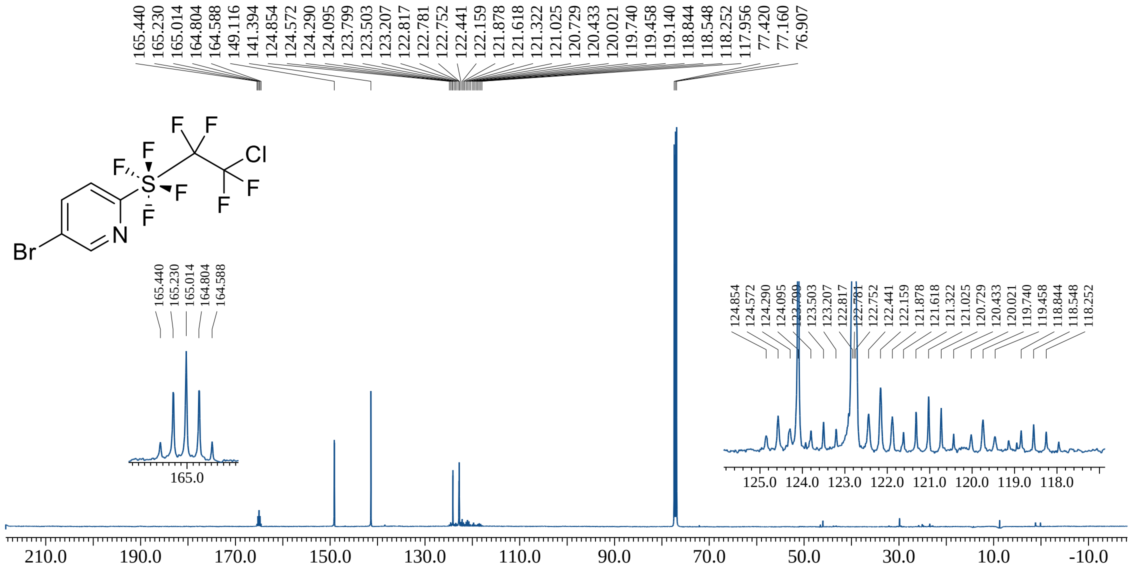

^{1}H NMR (700 MHz, Chloroform-d) of **9a**

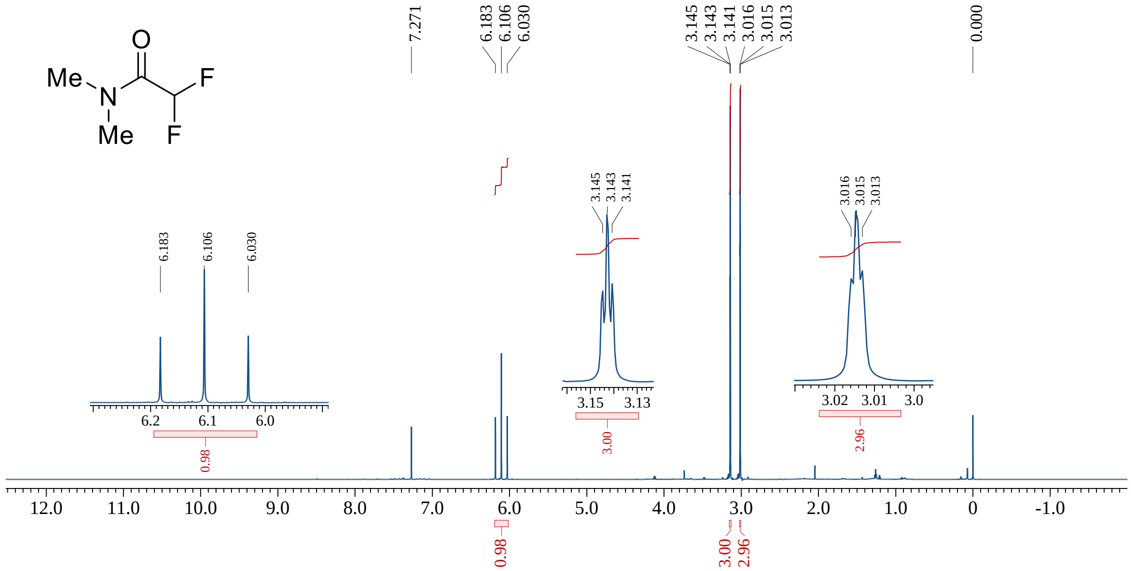

^{19}F NMR (282 MHz, Chloroform-d) of **9a**

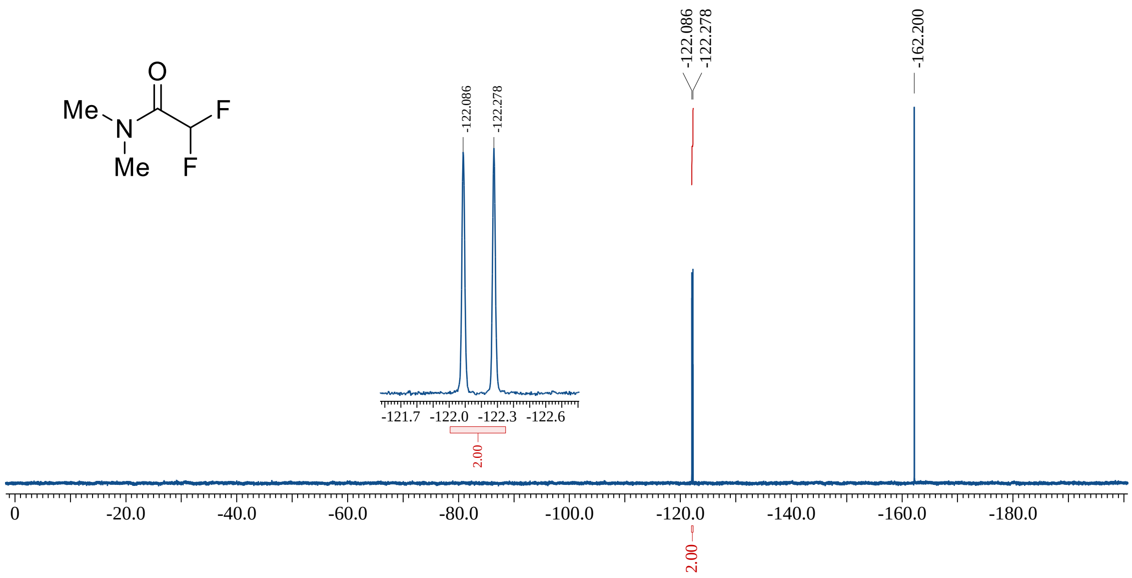

^{1}H NMR (300 MHz, Chloroform-d) of **9b**

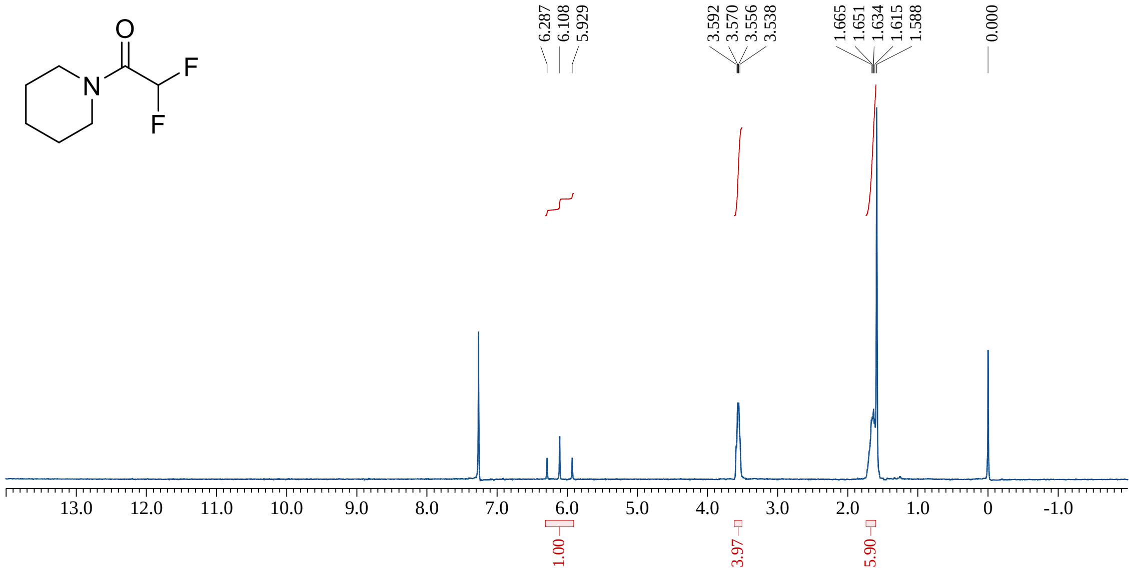

^{19}F NMR (282 MHz, Chloroform-d) of **9b**

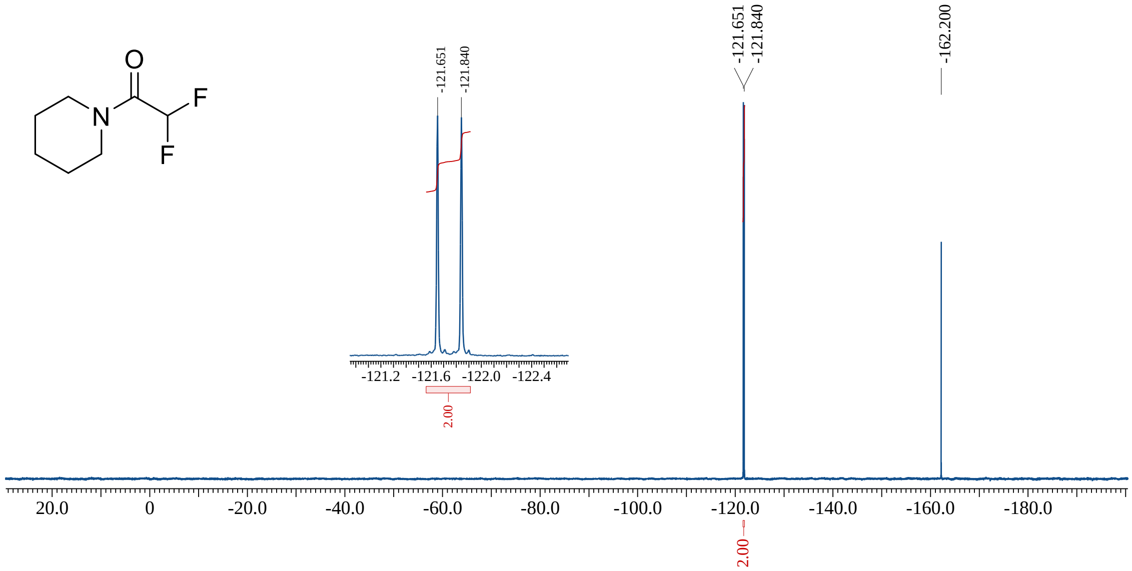

$^{13}C\ \{^{1}H\}$ NMR (126 MHz, Chloroform-d) of **9b**

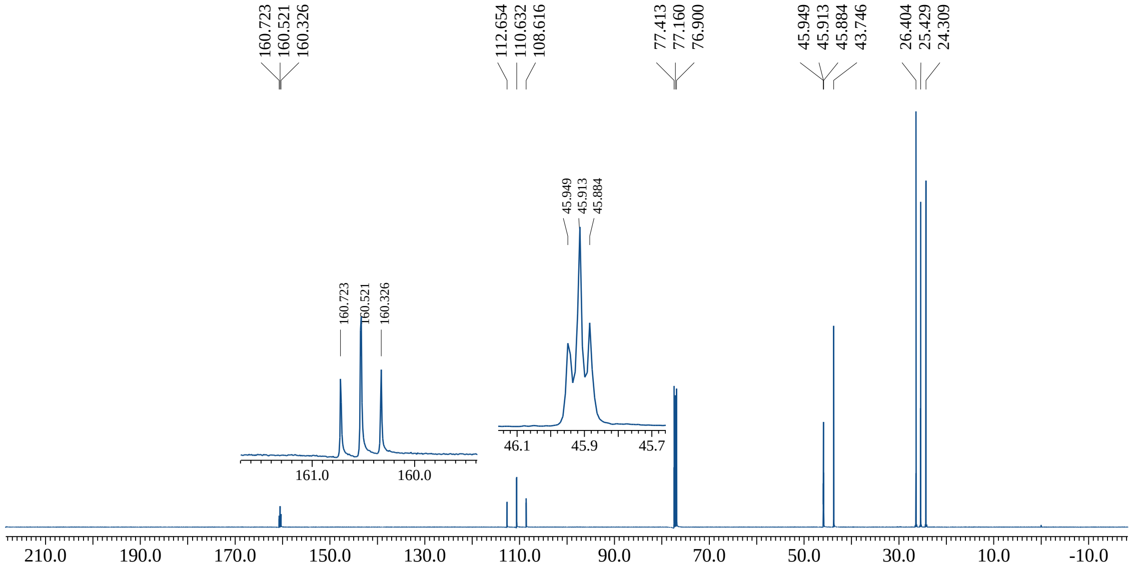

^{1}H NMR (300 MHz, Chloroform-d) of **11a**

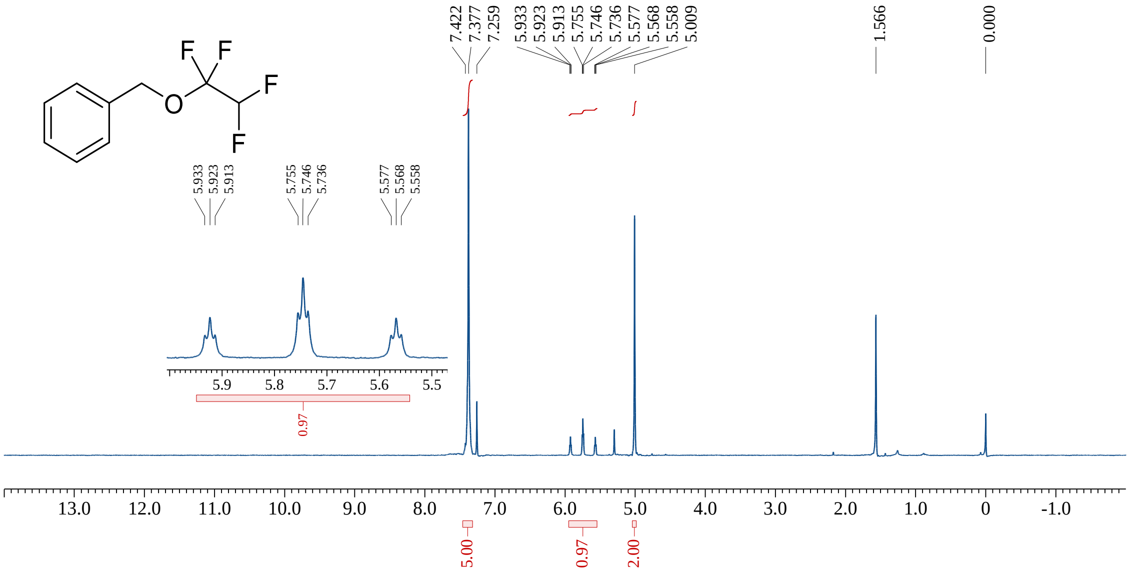

^{19}F NMR (282 MHz, Chloroform-d) of **11a**

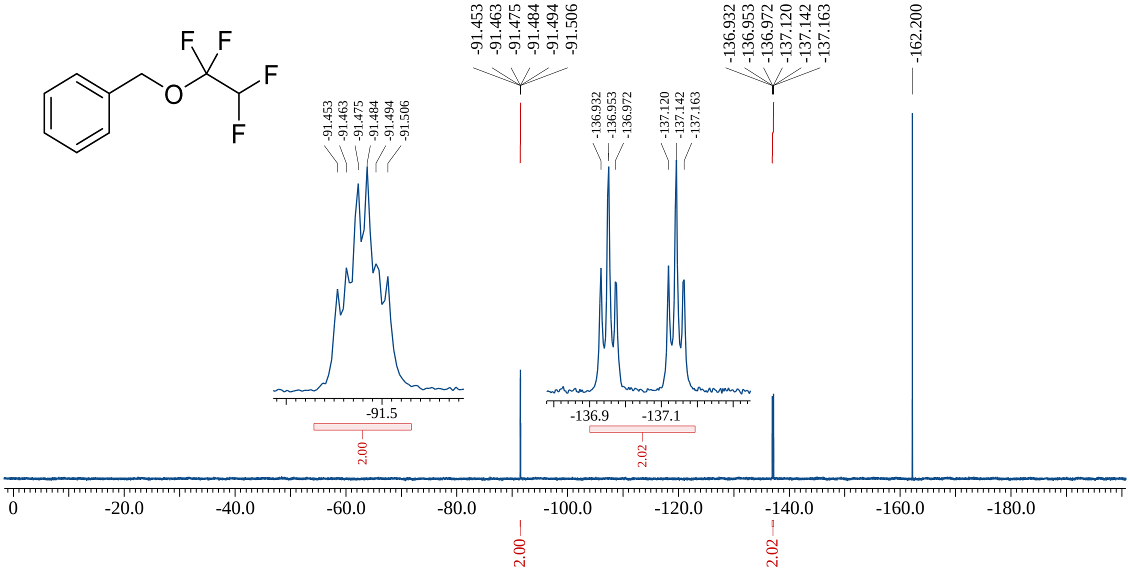

^{1}H NMR (300 MHz, Chloroform-d) of **11b**

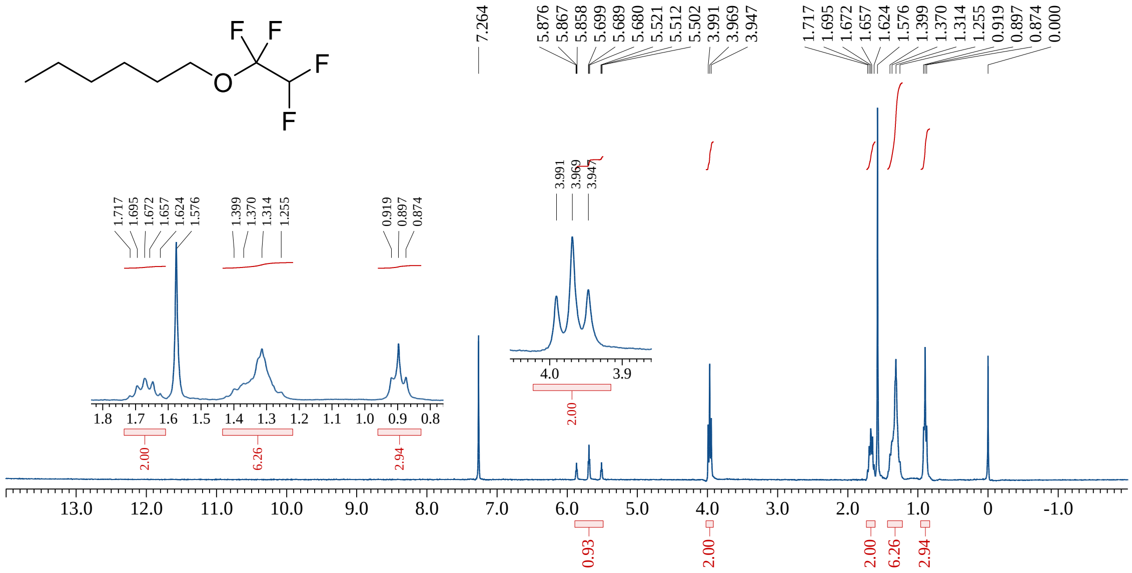

^{19}F NMR (282 MHz, Chloroform-d) of **11b**

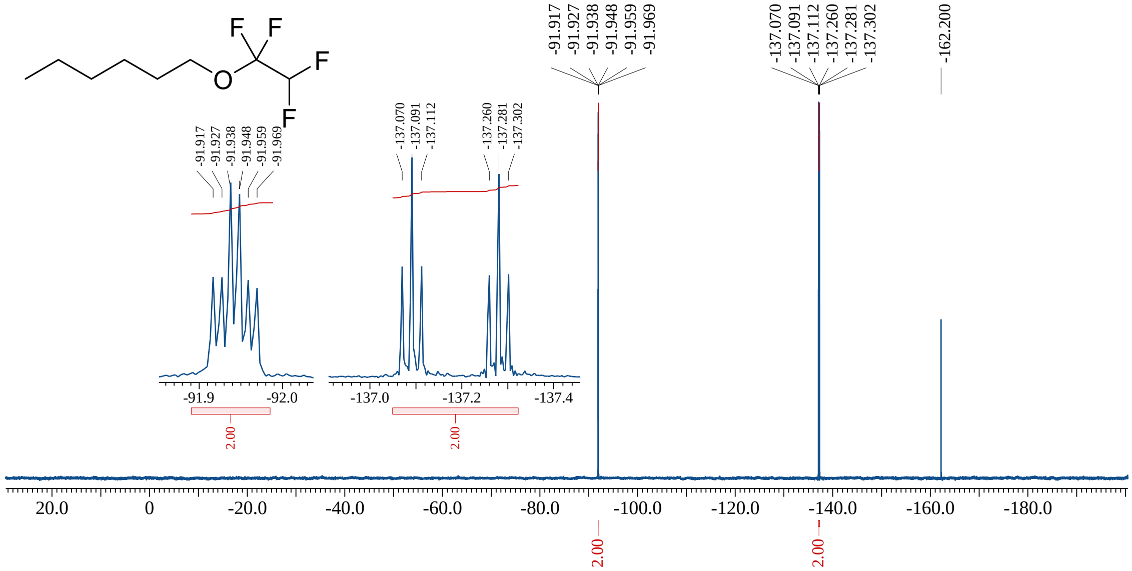

^{13}C {^{1}H} NMR (126 MHz, Chloroform-d) of **11b**

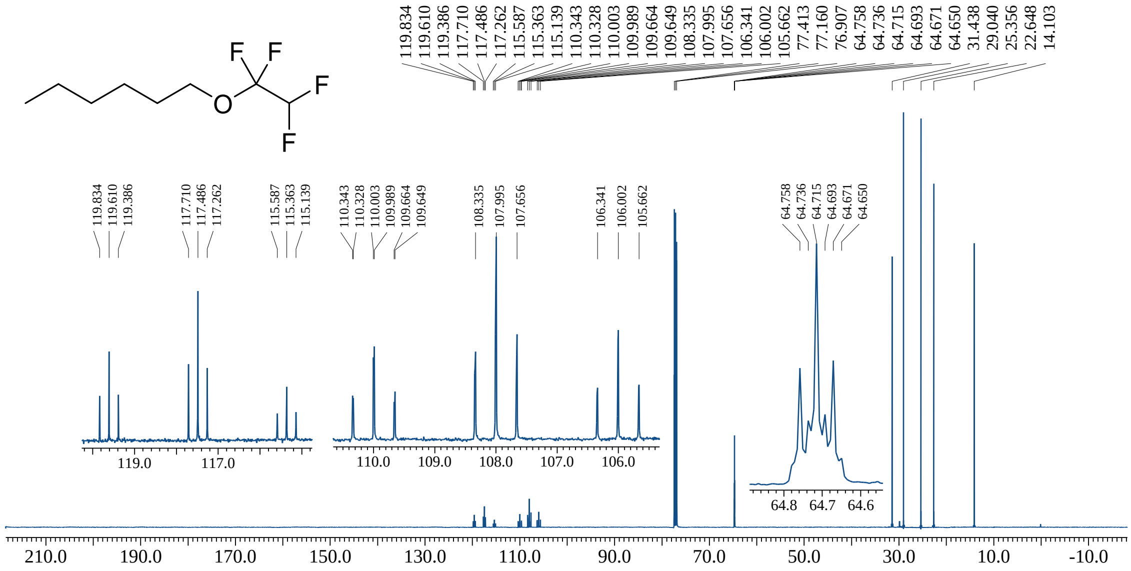

^{1}H NMR (600 MHz) of **12**

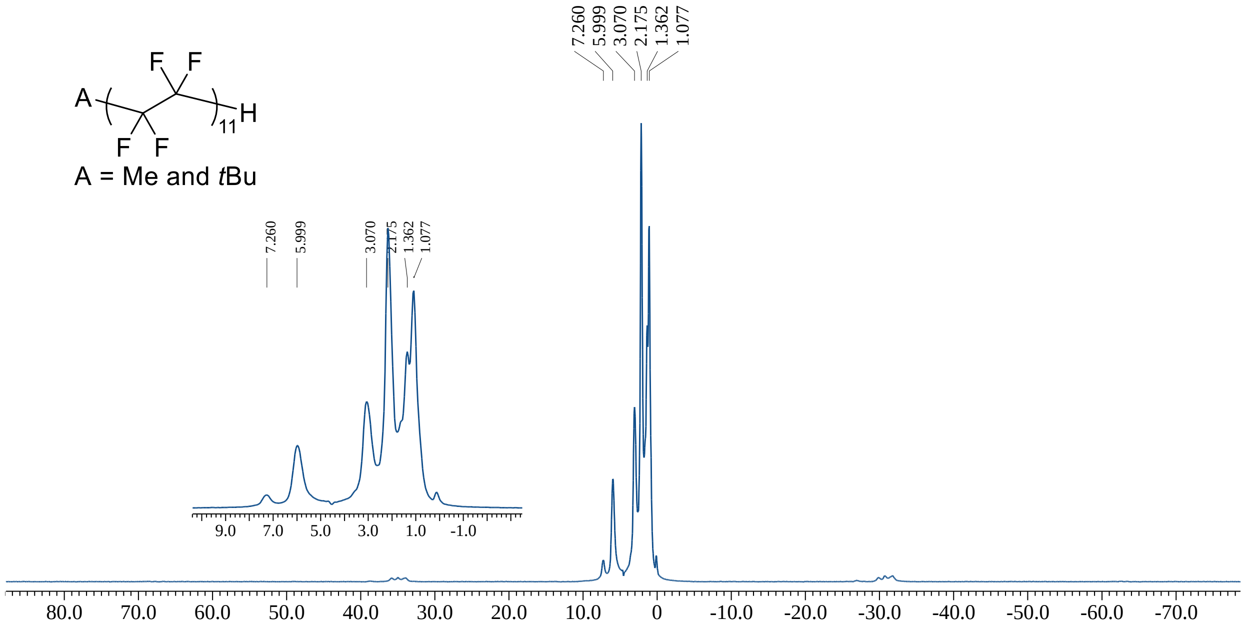

^{19}F NMR (565 MHz) of **12**

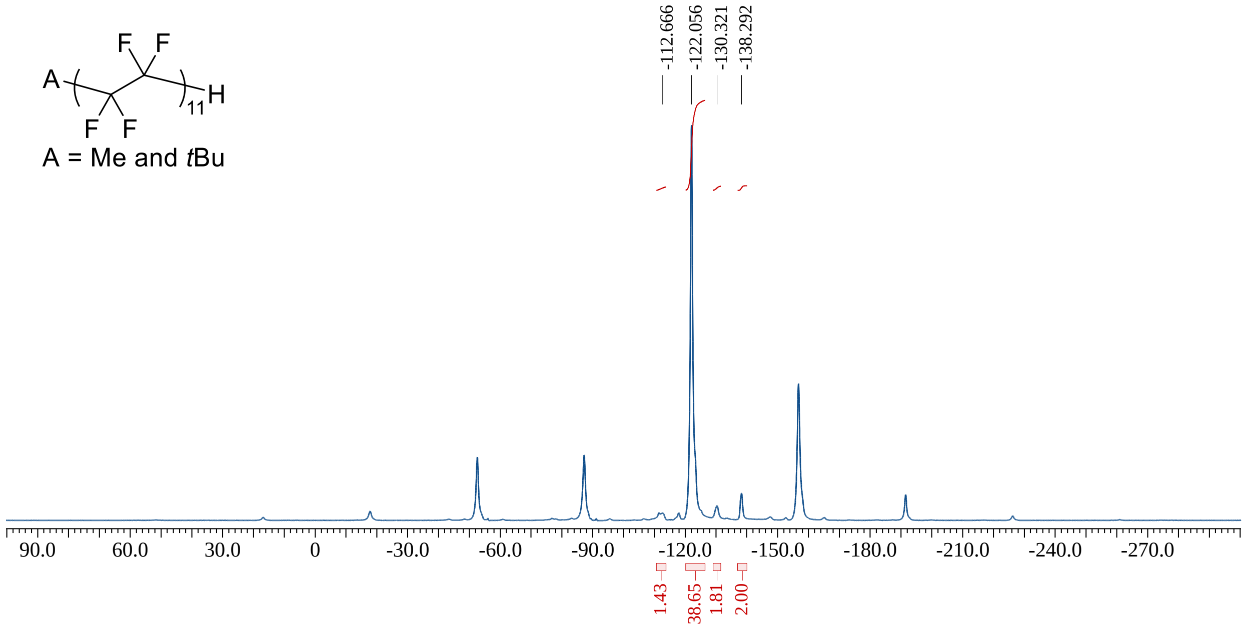

^{13}C {^{1}H} NMR (151 MHz) of **12**

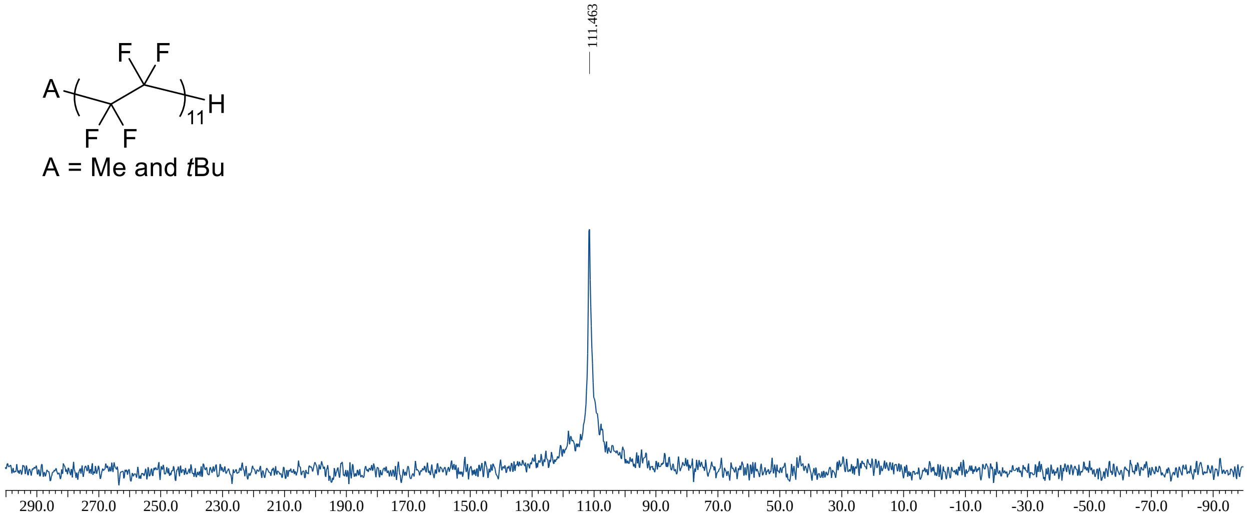

References

1. Frisch, M. J., Trucks, G. W., Schlegel, H. B., Scuseria, G. E., Robb, M. A., Cheeseman, J. R., Scalmani, G., Barone, V., Petersson, G. A., Nakatsuji, H., et al. *Gaussian 16, Revision B.01* (Gaussian, Inc., 2016).

2. Chai, J.-D. and H.-Gordon, M. (2008). Long-Range Corrected Hybrid Density Functionals with Damped Atom–Atom Dispersion Corrections. Phys. Chem. Chem. Phys. *10*, 6615-6620. https://doi.org/10.1039/B810189B.

3. Weigend, F. and Ahlrichs, R. (2005). Balanced Basis Sets of Split Valence, Triple Zeta Valence and Quadruple Zeta Valence Quality for H to Rn: Design and Assessment of Accuracy. Phys. Chem. Chem. Phys. *7*, 3297-3305. https://doi.org/10.1039/B508541A.

4. Marenich, A. V., Cramer, C. J. and Truhlar, D. G. (2009). Universal Solvation Model Based on Solute Electron Density and on a Continuum Model of the Solvent Defined by the Bulk Dielectric Constant and Atomic Surface Tensions. J. Phys. Chem. B *113*, 6378-6396. https://doi.org/10.1021/jp810292n.

5. Fukui, K. (1981). The Path of Chemical Reactions - the IRC Approach. Acc. Chem. Res. *14*, 363-368. https://doi.org/10.1021/ar00072a001.

6. Legault, C. Y. (2020). CYLview20. Université de Sherbrooke. http://www.cylview.org.
